# Full-length mRNA sequencing uncovers a widespread coupling between transcription initiation and mRNA processing

**DOI:** 10.1186/s13059-018-1418-0

**Published:** 2018-03-29

**Authors:** Seyed Yahya Anvar, Guy Allard, Elizabeth Tseng, Gloria M. Sheynkman, Eleonora de Klerk, Martijn Vermaat, Raymund H. Yin, Hans E. Johansson, Yavuz Ariyurek, Johan T. den Dunnen, Stephen W. Turner, Peter A. C. ‘t Hoen

**Affiliations:** 1 Department of Human Genetics, Leiden University Medical Center, Leiden, 2300 RC The Netherlands; 2 Leiden Genome Technology Center, Leiden University Medical Center, Leiden, 2300 RC The Netherlands; 3 Department of Clinical Pharmacy and Toxicology, Leiden University Medical Center, Leiden, 2300 RC The Netherlands; 4 Pacific Biosciences, 1305 O’Brien Drive, Menlo Park, CA 94025 USA; 5 Center for Cancer Systems Biology (CCSB) and Department of Cancer Biology, Dana-Farber Cancer Institute, Boston, MA 02215 USA; 6 Department of Genetics, Harvard Medical School, Boston, MA 02115 USA; 7 Department of Microbiology and Immunology, UCSF Diabetes Center, University of California San Francisco (UCSF), San Francisco, CA 94143-0534 USA; 8 LGC Biosearch Technologies, Petaluma, CA 94954-6904 USA; 9 Centre for Molecular and Biomolecular Informatics, Radboud Institute for Molecular Life Sciences, Radboud University Medical Center, Nijmegen, The Netherlands

## Abstract

**Background:**

The multifaceted control of gene expression requires tight coordination of regulatory mechanisms at transcriptional and post-transcriptional level. Here, we studied the interdependence of transcription initiation, splicing and polyadenylation events on single mRNA molecules by full-length mRNA sequencing.

**Results:**

In MCF-7 breast cancer cells, we find 2700 genes with interdependent alternative transcription initiation, splicing and polyadenylation events, both in proximal and distant parts of mRNA molecules, including examples of coupling between transcription start sites and polyadenylation sites. The analysis of three human primary tissues (brain, heart and liver) reveals similar patterns of interdependency between transcription initiation and mRNA processing events. We predict thousands of novel open reading frames from full-length mRNA sequences and obtained evidence for their translation by shotgun proteomics. The mapping database rescues 358 previously unassigned peptides and improves the assignment of others. By recognizing sample-specific amino-acid changes and novel splicing patterns, full-length mRNA sequencing improves proteogenomics analysis of MCF-7 cells.

**Conclusions:**

Our findings demonstrate that our understanding of transcriptome complexity is far from complete and provides a basis to reveal largely unresolved mechanisms that coordinate transcription initiation and mRNA processing.

**Electronic supplementary material:**

The online version of this article (10.1186/s13059-018-1418-0) contains supplementary material, which is available to authorized users.

## Background

The formation of a mature messenger RNA (mRNA) is a multi-step process. In higher eukaryotes, variations in each of these steps, including alternative transcription initiation, differential splicing of exons, and alternative polyadenylation site usage, change the content of the mature transcript. The multitude of transcripts arising from these events offers an enormous diversity of protein isoforms that can be produced from a single gene locus. Tight regulation and coordination of these processes ensures the production of a (limited) set of cell-, tissue-, and condition-specific transcript variants to meet variable cellular protein requirements [1–4]. Whether these processes are co-transcriptionally linked is currently largely unknown, as are the mechanisms that couple transcription with 5′ end capping, splicing, and 3′ end formation (reviewed in [5]). Thus, resolving full transcript structures and accurate quantification of the abundance of alternative transcripts are important steps towards the detection and understanding of these mechanisms.

RNA sequencing (RNA-seq) has become a central technology for deciphering the global RNA expression patterns. However, reconstruction and expression level estimation of alternative transcripts using standard RNA-seq experiments is limited and prone to error due to relatively short read length (typically up to 150 nt) and required amplification steps of second-generation sequencing technologies [6, 7]. It is apparent that single-molecule long reads that capture the entire RNA molecule can offer a better understanding of the rich patterns of alternative transcription initiation and mRNA processing events and, hence, the underlying biology.

Despite a number of studies that have pursued long read sequencing to connect different exons or even capture entire transcripts with a rather limited sequencing depth [6, 8–14], the coupling between transcription initiation and mRNA processing has not been extensively studied. Here, we investigate the global pattern of coupling between transcription initiation, splicing, and polyadenylation in MCF-7 human breast cancer cell line and three human tissues, which are deeply sequenced using the single-molecule real-time Pacific Biosciences RSII sequencing platform. We show that transcription initiation and mRNA processing are tightly coupled and that such interdependencies can be found across the entire RNA molecule and across large intra-molecular distances. We demonstrate that transcript identification and understanding of coupling between processes that are involved in the formation of these transcripts is far from complete, even in well-characterized human cell lines such as MCF-7. This study provides an in-depth view of the true complexity of the transcriptome and, for the first time, shows the tight and global interdependency between alternative transcription initiation, splicing and polyadenylation. We also show the value of this resource in relation to translation and sample-specific survey of the proteome.

## Results

### Detection and quantification of full-length transcripts in MCF-7 cells

To investigate the genome-wide coupling of transcription initiation and mRNA processing, full-length mRNAs from MCF-7 human breast cancer cells were sequenced on 147 SMRT cells using the Iso-Seq method on the Pacific Biosciences RSII platform (Additional file 1: Table S1). Before sequencing, parts of the sequencing library were size selected to allow for a good representation of longer transcripts.

**Fig. 1:**
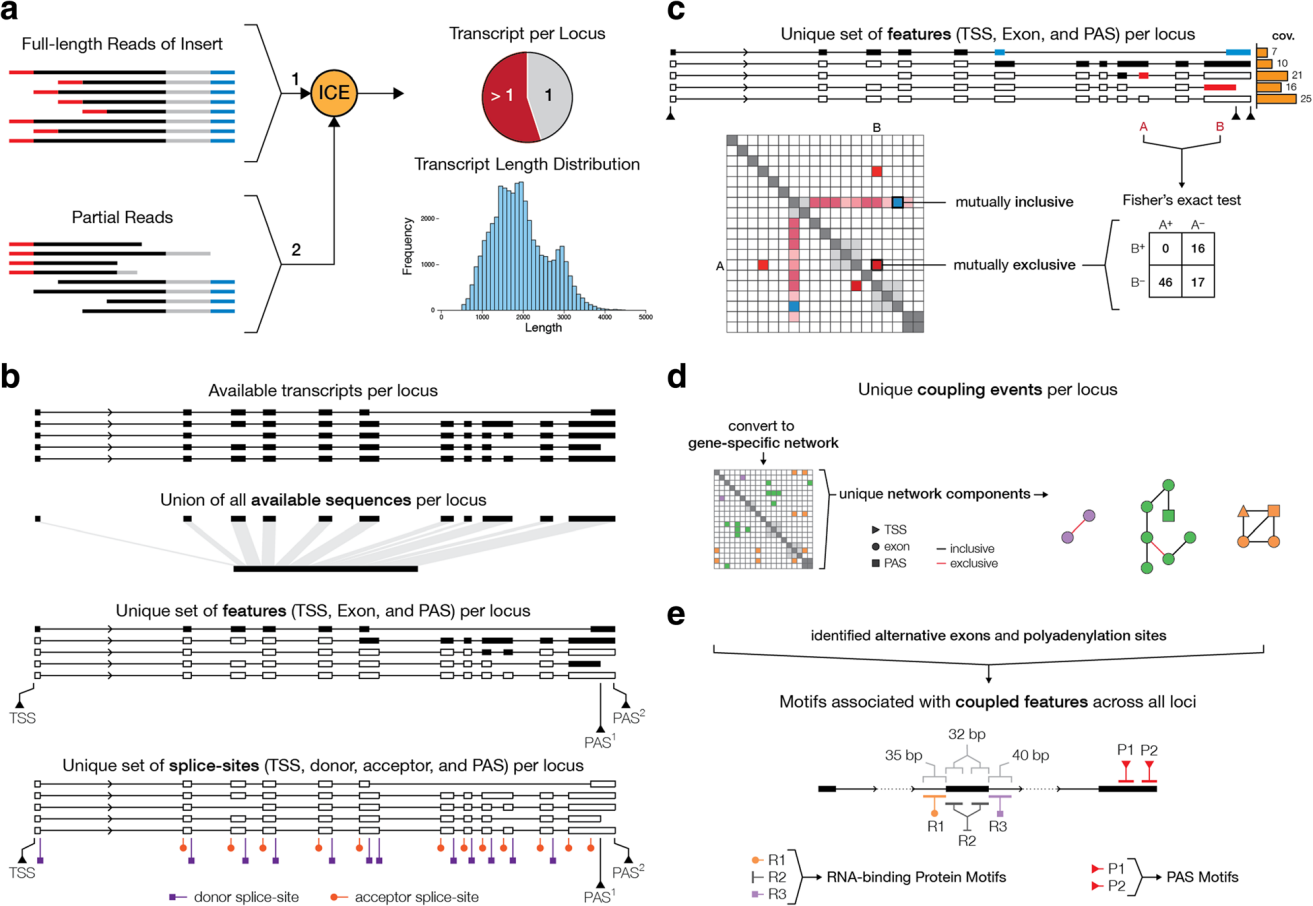
*Schematic* overview of the approach to characterize the interdependencies between mRNA transcription initiation and processing events. **a** Identified full-length reads (reads with RNA inserts between 5′ and 3′ primers) are clustered into unique transcript structures using the ICE algorithm and further polished using the partial reads, where one of the primer sequences is missing. **b** Based on available transcripts per locus, available sequence (union of all exonic sequences that are observed at each locus) and unique set of features and splice sites are identified. Feature sets comprise unique transcriptional start sites (TSS), exons, and polyadenylation sites (PAS). The unique set of splice sites consists of unique donor and acceptor splice sites as well as all alternative TSSs and PASs. **c** The survey of coupling events is done by performing all possible pairwise tests between unique features in genes. The sum of the coverage of all transcripts that support the inclusion or exclusion of each pair is used in a contingency table to perform a Fisher’s exact test for statistical significance. The odds ratio (OR) is used to differentiate between mutually inclusive and exclusive coupling. **d** Set of interdependent coupling events were identified based on networks of coupling between features in each gene. Nodes represent features and links depict the mutual inclusivity (*black edges*) or mutual exclusivity (*red edges*) of each feature pair. Unique network components can thereby be filtered based on the type of interaction: mutual inclusive or mutual exclusive coupling events. **e** For all alternative exons that show significant coupling, a motif search is performed to assess the enrichment of specific RNA-binding protein motifs. For all alternative exons, 35-bp intronic sequences upstream of the acceptor site are defined as R1 domain (depicted in *orange*), 32-bp exonic sequences downstream of the acceptor site and upstream of the donor site are defined as R2 domain (depicted in *dark gray*), and 40-bp intronic sequences downstream of the donor site are defined as R3 domain (depicted in *purple*); 35-bp sequence upstream of each PAS (depicted in *red*) is searched for the presence of canonical and non-canonical poly(A) signals

Transcript structures were defined by applying the isoform-level clustering algorithm (ICE) on full-length reads [15], capturing the entire mRNA molecule (containing both 5′ and 3′ primer sequences). Final consensus sequence of each transcript cluster was obtained by using both the full-length and associated partial reads (Fig. 1a). The analysis pipeline precisely determined the position of polyadenylation sites (presence of poly(A) tail in the sequence) and intron-exon boundaries, as evident from the presence of the canonical GU motif in 93% of donor splice sites and the canonical AG motif in 95% of acceptor splice sites. In fact, 90% of introns were defined by canonical splice-site motifs (GU-AG). In addition to 7364 single-exon transcripts, the MCF-7 transcriptome consists of 11,350 multi-exon genes, of which 69% produced multiple transcript structures (Additional file 1: Figure S1). Multi-exon transcripts range from 54 bp (matching *ANKRD36–004* transcript) to 10,792 bp (matching *TAX1BP1* gene with novel splice junctions) in length with an average of 82 supporting reads (Additional file 1: Figure S1). Moreover, 49% of identified transcripts in MCF-7 were found to be potentially novel in comparison with the Gencode annotation (Additional file 1: Table S2). In case of single-exon transcripts, the majority (78.7%) were located within genes. However, we could find a Gencode match for only 9.2% of single-exon transcripts in our dataset. This may be due to the fact that resolution of single-exon transcripts with classical experiments and standard RNA-seq approaches is very challenging and, therefore, it has resulted in underrepresentation of such mRNA molecules from current annotations of many vertebrates [16].

To support the qualitative and quantitative accuracy of the analysis pipeline, the measured gene expression levels were compared to those obtained from five publicly available RNA-seq MCF-7 datasets generated on Illumina HiSeq2000 or HiSeq2500 platforms. Spearman correlations between Iso-Seq and standard RNA-seq were in the range of 0.69–0.75 (Additional file 1: Figure S2A). Differences in library preparation protocols, presence of fewer duplicates, and uniformity of coverage in the PacBio data as well as contrast in sequencing dynamics contribute to minor differences observed in estimated gene expression levels [17]. Although we observed some inter-dataset differences in detected genes, for most genes, the results from all datasets were in concordance (Additional file 1: Figure S2B). Transcript lengths and loading bias did not significantly contribute to inter-platform differences for gene detection as similar results were also found for intra-platform comparisons (Additional file 1: Figure S2C). Thus, the full-length mRNA sequencing data can be reliably used for locus-specific quantification in MCF-7 cells.

To further establish the accuracy of the transcriptional start sites (TSSs) and polyadenylation sites (PASs) obtained with the Iso-Seq method, we performed a transcriptome-wide comparison of 5′ and 3′ ends of full-length transcripts with those found in Encode CAGE and RNA-PET experiments in MCF-7 cells. The majority of TSSs detected by full-length mRNA sequencing were in close proximity to their counterpart in the Encode CAGE dataset, with a median distance of 1 bp (Additional file 1: Figure S3A). In addition, 99.5% of genes with multiple TSSs in the CAGE dataset were also represented by multiple TSSs in PacBio data. We could identify 1386 additional multi-TSS genes using full-length mRNA sequencing. The Encode RNA-PET dataset captures the connections between 5′ and 3′ ends of transcripts. These data were used for the validation of the combinations of TSSs and PASs in the transcript structures identified by full-length mRNA sequencing in MCF-7 cells. However, it is important to note the limited resolution of RNA-PET data as 5′ and 3′ tags that were located within 100 bp of another tag were merged into a single cluster. The results of this comparison further confirm the validity of detected TSSs and PASs of identified transcripts as the majority of RNA-PET terminal sites were in close proximity of their counterparts in Iso-Seq data, with median distance of 70 bp and – 11 bp, respectively (Additional file 1: Figure S3B). Overall, these results provide an independent confirmation that reported transcripts are most likely full-length and no systematic bias is introduced during the full-length complementary DNA (cDNA) synthesis and computational resolution of mRNA sequences (Additional file 1: Figure S3C).

### Identification of interdependencies between transcription initiation, splicing, and polyadenylation

To detect and characterize the dependency between transcription initiation and mRNA processing events, we designed the following analysis strategy (Fig. 1): for each gene, the union of all exonic sequences was considered as the available sequence and the union of all unique transcriptional start sites, exons (defined as having distinct donor and acceptor splice sites), and polyadenylation sites was used as a set of available features (Fig. 1b). Interdependency between all valid combinations of features (alternative TSSs, exons, and PASs) was assessed based on the number of reads that support the preferential inclusion or exclusion of each pair of features (Additional file 1: Figure S4). The following criteria were used to assess interdependency between meaningful pairs of features: (1) only multi-transcript loci were examined; (2) single-exon transcripts were excluded from the analysis; (3) two features should not partially or fully overlap; (4) dependency between alternative TSSs and features located in their upstream region were omitted as their exclusivity is given (the same rule was applied to downstream of alternative PASs); and (5) only transcripts that fully encompass the region represented by two features were used to populated the two-by-two contingency table. To test preferred mutual inclusion/exclusion of each pair of features, we applied a Fisher’s exact test followed by Bonferroni multiple testing correction to evaluate statistical significance of the interdependency (Fig. 1c; also see “Methods”). Since features may be coupled to a few other features, the actual coupling events were summarized into a series of network components within a gene-specific interaction network to capture the independent coupling events (Fig. 1d). These components can be summarized based on the level of connectivity or mutual inclusivity or exclusivity within each network to construct subnetworks. We subsequently searched the sequences containing the coupled alternative exons or poly(A) sites for enriched sequence motifs and tested whether they contain motifs of known RNA-binding proteins (RBPs) (Fig. 1e).

### General properties of coupling in human MCF-7 transcriptome

The MCF-7 transcriptome consists of 11,350 multi-exon genes that present 3,532,796 combinations of features (TSSs, exons, and PASs), mainly representing exon–exon pairs as single TSS or PAS was found in many loci (Additional file 1: Figure S5). After initial filtering, 7708 genes and 3,055,099 pairs of features were considered for statistical analysis of their interdependence.

**Fig. 2:**
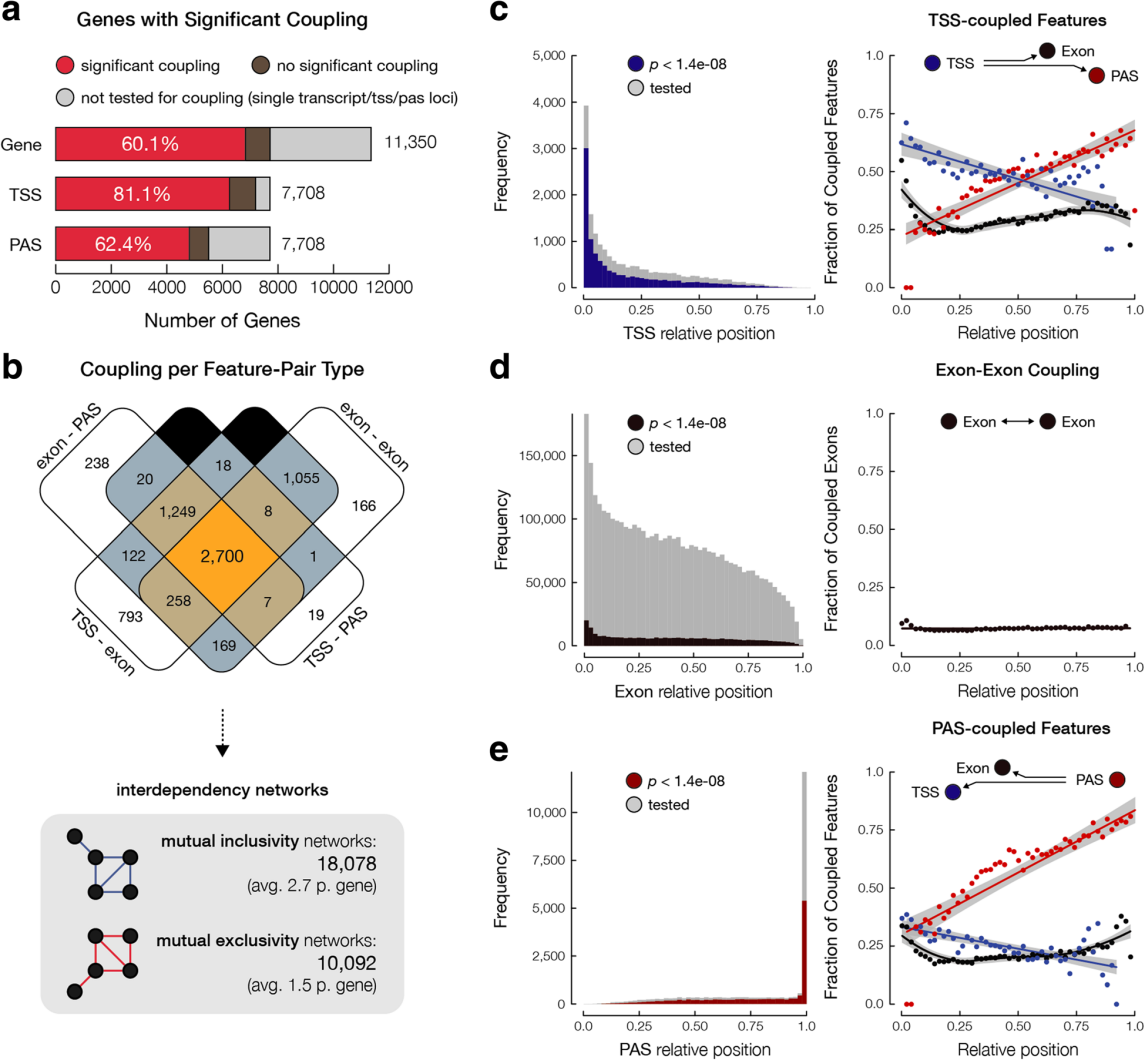
Alternative transcription, splicing, and polyadenylation are highly interdependent. **a** *Bar charts* illustrate the number and proportion of genes that show significant coupling in MCF-7 cells. Genes with TSS- or PAS-coupled features are also presented. **b** *Venn diagram* shows the number of genes with various types of coupling representing interdependencies between different alternative processes. The total number of mutually inclusive and exclusive networks are also listed. **c** *Histogram* of the relative positions of TSSs with (*blue*) and without (*gray*) significant coupling to mRNA processing events. Relative positions are calculated based on the length of the total exonic sequence at each locus. *Scatter plot* shows the fraction of significantly coupled TSSs (*blue*) to alternative exons (*black*) and PASs (*red*), plotted at each relative position. **d** *Histogram* of the relative positions of alternative exons with (*brown*) and without (*gray*) significant coupling to other exons. *Scatter plot* shows the fraction of significantly coupled exons to other exons, plotted at each relative position. **e** *Histogram* of the relative positions of PASs with (*red*) and without (*gray*) significant coupling to alternative transcription and splicing events. *Scatter plot* shows the fraction of significantly coupled PASs (*red*) to alternative TSSs (*blue*) and exons (*black*), plotted at each relative position. For plots depicting the percentage of linked features per position, the bin size of 0.02 was used

Almost 10% of all feature pairs were significantly coupled (*p* value < 1.4e-08, after Bonferroni correction for multiple testing). We observed almost equal distribution over mutually inclusive (52%) and mutual exclusive pairwise interdependencies (Additional file 1: Figure S6A). Notably, we found coupling between mRNA features in over 60% of all multi-exon genes (6825 out of 11,350; Fig. 2a), represented by 18,078 mutually inclusive and 10,092 mutually exclusive subnetwork components (Fig. 2b). Particularly, alternative TSSs appear to have a significant impact on mRNA processing as > 80% of multi-transcript genes exhibit interdependency between the choice of TSS and alternative splicing. Of the 6825 genes with at least one coupling event, 2700 (37%) showed interdependencies between all classes of features (Fig. 2b; Additional file 1: Figure S6A): alternative TSS linked to alternative exons, alternative exon to alternative exon linkage, alternative PAS linked to alternative exons, and alternative TSSs to alternative PASs. Thus, the deep sequencing of full-length mRNAs provided a first image of the large degree of coordination in the usage of alternative TSSs, exons and PASs, restricting the number of produced transcripts given the substantial amount of combinatorial possibilities.

The length of individual transcripts was not associated with the likelihood of a significant coupling event in that transcript (Additional file 1: Figure S6B). However, after examining the length of the union of exonic sequences per gene and the likelihood of observing coupling, we found that significant coupling events were enriched in genes with larger available exonic sequences (Additional file 1: Figure S6B). As expected, a larger exonic region in each locus gives rise to a larger repertoire of possible transcripts, requiring more extensive regulation of the synthesis for transcripts containing different subset of features.

To decipher whether observed interdependencies primarily occur between proximal features, we also examined the effect of the relative position in the gene and the distance between features on the observed degree of coupling. As expected, most TSSs were located at the most 5′-end of genes. However, interdependency of alternative TSSs was observed across the entire gene (Fig. 2c, left panel). Alternative TSSs were preferentially coupled to alternative splicing events in relatively close proximity to the TSSs, near the 5′-end, as well as distal exons at the 3′-end (Fig. 2c, right panel). Examples of the coupling of alternative TSS and alternative exon usage across large distances, and spanning multiple exons were frequently observed (Additional file 1: Figure S7,8; *ALDOA* and *C1QTNF6*). More evidence for interactions across the entire length of genes comes from the significant coupling between TSS and PAS, the two most distant features in an mRNA molecule (Additional file 1: Figure S9; *NCAPD2*).

Dependencies between multiple alternative splicing events were uniformly observed across the entire gene (Fig. 2d; Additional file 1: Figure S10, *LMNA*). Despite the uniform distribution of exon–exon coupling events and the presence of distant coupling events (Additional file 1: Figure S11, *RELA*), most interdependent alternative splicing events were between nearby or neighboring exons (Additional file 1: Figure S12, *CALU*).

Like alternative TSSs, coupling events linked to alternative PAS usage were found across the entire gene (Fig. 2e). In concordance with published literature [18–20], alternative PAS usage was preferentially coupled to nearby alternative exons (Fig. 2e). Nevertheless, many alternative PASs were coupled to alternative exons proximal to 5′-regions of genes.

We performed Sanger sequencing to independently verify identified coupling events for a set of gene loci. Due to limited range of alternative technologies such as Sanger sequencing, only relatively close coupling events could be assessed. In most cases, the Sanger sequencing results were in full concordance with the coupling events identified using full-length mRNA sequencing (Additional file 1: Figure S7, 8, 10, and 11). In addition, we carried out a single-molecule RNA in situ fluorescence (smRNA FISH) co-localization approach [21, 22] to examine the alternative splicing events that were identified by full-length RNA-seq and confirmed by Sanger sequencing. Four probe sets were designed to detect different segments of the *CALU* mRNAs at single-molecule level. The full CALU_E probe set covers the common exons on the full-length variants, whereas the 9-oligo CALU_E4 and CALU_E5 sets were designed to specifically hybridize to either exon 4 or 5. The signal from the common exon probes was easily detected (Additional file 1: Figure S12) and could be used for co-localized signals from the exon 4, exon 5, and intron 1 sets. The average numbers and distribution of signals revealed that cytoplasmic mRNAs predominantly either include exon 4 (~ 50 copies per cell) or exon 5 (~ 10 copies per cell), followed by mRNAs that do not include exon 4 and 5 (~ 9 copies per cell). The co-localized signals from all three exon sets and all four sets were exclusively located in the nuclear domain with much lower abundance (Additional file 1: Figure S12). This is consistent with active *CALU* gene transcription bursts that contain pre-mRNAs in various states of post-transcriptional processing and thus expected to contain common exons, alternative exons, and introns. In short, by applying RNA FISH, we could reveal the identity and distribution of single mRNA molecules of *CALU* as well as independently confirm the mutual exclusivity of exon 4 and 5 in MCF-7 cells. Together, the results of Sanger sequencing and RNA FISH experiments are in concordance with full-length RNA-seq and support the coupling events identified.

### Poly(A) signal usage for coupled polyadenylation sites

Most alternative PASs in MCF-7 cells were found in tandem (in the same terminal exon, generating a longer or shorter 3’-UTR). From 5498 genes with multiple PASs, we identified 10,927 tandem PASs in the same exon across 3983 genes (72%). From these, 3465 loci (87%) included PASs that were significantly coupled with alternative TSSs or alternative exons. Still, many coupling events between alternative PASs and inclusion or exclusion of alternative exons were due to the use of exonic and intronic PASs (8171 non-tandem PASs), leading to the formation of new 3’ UTRs.

**Fig. 3:**
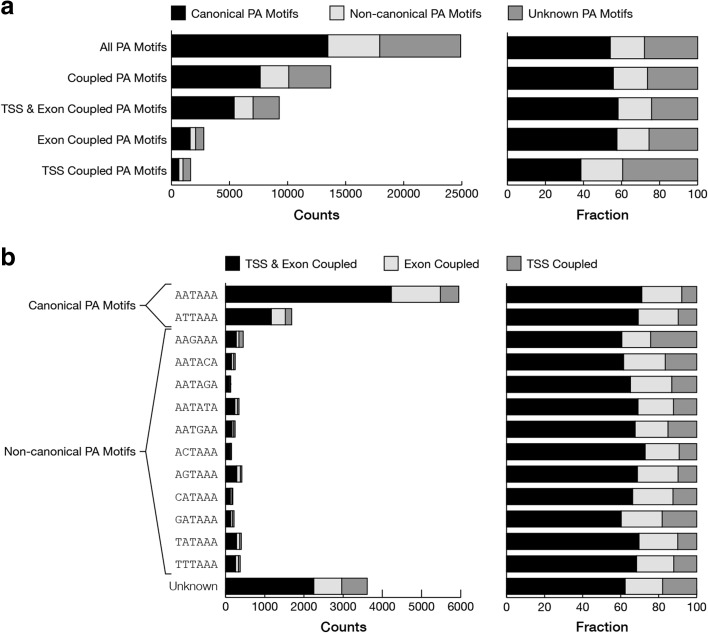
Alternative TSSs and exons are significantly associated with known and novel poly(A) signals. **a** *Bar charts* show the number and relative proportion of PASs that are associated with canonical or non-canonical poly(A) signals for all PASs, PASs with significant coupling, and alternative exon- and/or TSS-linked PASs. **b** *Bar charts* represent the number and relative proportion of known and unknown poly(A) signals for TSS-linked, exon-linked, or TSS- and exon-linked PASs

To assess whether certain poly(A) signals are preferentially associated with alternative transcription and splicing, we searched for canonical (AATAAA and ATTAAA) and 11 known non-canonical poly(A) signals in the 35-bp sequences upstream of the identified PASs. Canonical and known non-canonical poly(A) signals could be found in the 35-bp sequences upstream of 54% and 18% of all PASs, respectively (Fig. 3a). The proportion of PASs that could be associated with canonical poly(A) signals was unchanged (55.7%) for those that were coupled with TSSs or alternative exons. However, PASs that were linked with TSSs showed a lower proportion of canonical poly(A) signals (38.6%). Although this decrease was accompanied by a slight increase in known non-canonical poly(A) signals, it was mainly due to the use of alternative PASs for which no known poly(A) signal could be found (Fig. 3a, b). This suggests that novel poly(A) signals and other mechanisms may be involved in transcription-coupled polyadenylation in MCF-7 cells. Thus, we screened for enriched motifs in the 35-bp sequences upstream of PASs that were not associated with known poly(A) signals. Based on a de novo motif enrichment analysis, we identified the enrichment of AKCCTGG for PASs with unknown poly(A) signal (Table 1). This motif was also significantly enriched in PASs that were coupled with alternative TSSs or splicing. Interestingly, this motif could be associated with the binding site of muscleblind-like (MBNL) protein family, known to play a dual role in the regulation of splicing and polyadenylation [23, 24]. Each MBNL isoform can bind to slightly different motifs [24] and a few motifs have been previously associated with MBNL proteins [24–26]. Although all three MBNL proteins are expressed in MCF-7 cells, the enrichment of de novo identified AKCCTGG and the recently reported CWGCMWKS motifs (mainly recognized by MBNL3 protein [24]) were more prominent. Additionally, previously identified binding motifs for MBNL1 (CTSCYB [25] and RSCWTGSK [24]) and MBNL2 (TGCYTSYY [24]) were also enriched in sequences upstream of the PASs without a known poly(A) signal (Table 1). However, these motifs were not found to be preferentially associated with PASs that were coupled with alternative TSSs or alternative exons. Together, these results suggest that MBNL proteins may play a role in mediating alternative splicing and alternative polyadenylation.

**Table 1:** Enrichment of MBNL binding site motifs in sequences upstream of alternative PAS with unknown poly(A) signal that are coupled with alternative TSS or alternative exons

Motifs	Source	Total	Random set	*p* value ^a^	Coupled PAS	Not coupled PAS	*p* value ^b^
AKCCTGG	DREME	1271	35	0	881	390	9.8E-44
CTSCYB	Masuda, 2012 [25]	898	708	2.6E-07	442	456	9.7E-01
YGCY	Purcell, 2012 [26]	2961	3139	1.0E-00	1578	1383	3.2E-02
RSCWTGSK	Batra, 2014 [24] – MBNL1	145	93	4.1E-04	80	65	2.5E-01
TGCYTSYY	Batra, 2014 [24] – MBNL2	95	55	6.5E-04	50	45	4.9E-01
CWGCMWKS	Batra, 2014 [24] – MBNL3	1306	139	4.7E-262	870	436	1.5E-32
	Total PASs	6979	6979	–	3614	3338	–

^a^The enrichment of binding motifs in sequences upstream of PASs without a known poly(A) signal were calculated by Fisher’s exact test (one-sided). A randomly generated set was used as a background for enrichment analysis

^b^PASs without significant coupling were used as the background set to identify a binding site that is enriched in the coupled PASs without a known poly(A) signal

### Identification of binding motifs for RNA-binding proteins potentially involved in coupling

We examined the potential involvement of RBPs in the coordination of alternative transcription initiation and mRNA processing events by enrichment analysis of their binding motifs in coupled vs non-coupled exons (background set). We screened three genomic domains relative to donor and acceptor splice sites of coupled exons for enriched sequence motifs (Fig. 1e; also see “Methods”): the 35-bp intronic sequences upstream of the acceptor site (R1), the 32-bp exonic sequences downstream of the acceptor sites and upstream of the donor sites (R2), and the 40-bp intronic sequences downstream of the donor sites (R3).

For coupled non-terminal exons, the sequences from the R1 domain (upstream of the acceptor) were enriched for motifs (Table 2) that can be recognized by the splicing modulators RBM24 [27] and SAMD4A [28] proteins. In addition, the R2 sequences were enriched for binding sites of RBM4B [29], NOVA2 [30, 31], and RBM28 [32] proteins, known to play a role in regulating alternative splicing. In fact, many RBM proteins have been associated with pre- and post-mRNA splicing events. R3 regions (downstream of the donor splice sites) were also enriched for motifs associated with alternative splicing modulators: FUS [33–35]; SRSF2 [36]; RBM5 [37–39]; PCBPI1; and PCBPI2 [40, 41] (Table 2). Together, we observed a clear indication that RBPs involved in regulation of alternative splicing and mRNA stability are likely to play a role in preferential selection of alternatively spliced exons.

**Table 2:** The RNA-binding protein motifs associated with alternative exons that are coupled to TSS, other alternative exons, or PAS

R1 domain						
Motif	Length	Coupled (28,716)	Not coupled (70,336)	E-value	Pfam ID	RBP
SVGV	4 nt.	12,121	23,872	6.0E-127	PF00536	SAMD4A
TGTCTGAA	8 nt.	108	70	1.2E-014	PF00076	RBM24; ENOX1
R2 domain						
Motif	Length	Coupled (53,490)	Not coupled (131,953)	E-value	Pfam ID	RBP
GSSB	4 nt.	29,261	65,661	2.4E-078	PF00076; PF00098	RBM4B
GGGAYTAC	8 nt.	223	164	2.8E-027	PF00013	NOVA2
AGTMGCT	7 nt.	262	234	2.8E-024	PF00076	RBM28
R3 domain						
Motif	Length	Coupled (28,591)	Not coupled (70,138)	E-value	Pfam ID	RBP
SGTRAG	6 nt.	1043	1330	7.6E-051	PF00076; PF00641 PF00013	FUS; SRSF2 PCBP1; PCBP2
GAAGGTGA	8 nt.	98	49	1.5E-016	PF00076; PF00641	RBM5

### Conservation of interdependencies across human tissues

We investigated whether the interdependent transcription initiation, splicing, and polyadenylation events identified in MCF-7 cancer cells could also be found in the full-length transcriptomes of three primary human tissues: brain; heart; and liver. As the sequencing depth in the primary tissues was lower than that of MCF-7, we could only examine the relatively abundant genes. In the human brain, of 5381 genes that could be assessed for coupling between transcription initiation and mRNA processing, 30.7% were found to have at least one coupling event (Additional file 1: Figure S13A). In total, we identified 7% of 789,054 possible combinations to be significantly interdependent. Similar patterns could be found in heart and liver, having 25% and 26% of genes exhibiting at least one coupling event, respectively (Additional file 1: Figure S13A). Pairwise comparison of coupling rates between transcriptomes revealed that the proportion of genes exhibiting coupling in multiple samples show a modest range of 5–13%, whereas for multi-transcripts genes, a larger proportion (in the range of 24–40%) exhibit at least one coupling event (Additional file 1: Figure S13B). Overall, MCF-7 transcriptome showed the largest gene overlap with other datasets suggesting that by achieving a deeper coverage many more interdependencies may be found that are conserved between tissues.

Next, we assessed the conservation of individual coupling events in different samples. From the total number of feature pairs that were found to be interdependent in at least one sample, by far the majority were found to be specific to a given tissue or MCF-7 cells since only 6–14% were found to be coupled in two examined datasets (Additional file 1: Figure S13C). Interestingly, feature pairs that were found to be interdependent in two samples were generally (~ 77%) mutually inclusive or exclusive in both tissues (Additional file 1: Figure S13C). This observation suggests that although most coupling events are tissue- or condition-specific, there seems to be a set of interdependencies that are conserved across multiple human tissues.

Finally, we performed a survey of Gencode annotation to identify the number of preferentially interdependent alternative exons that are already reflected in annotated transcripts. To do this, for all coupled exons, locus-specific relative number of transcripts that support the inclusion of two alternative exons was compared to the relative number of transcripts that only contain one of the two. Importantly, only Gencode transcripts that fully encompass the coupled exons were included in the analysis. In MCF-7 cells, > 91% of alternative exons could be found in the Gencode, from which the majority were mutually inclusive (Additional file 1: Figure S14A). The majority of alternative exons that were found to be mutually exclusive (87%) also seem to only occur independently in the Gencode annotation, whereas for those that were found to be mutually inclusive the concordance was weaker (58%). Nevertheless, in almost 79% of the cases, we found over twofold more incidences of mutually inclusive exons being annotated in the same transcript than independently. Overall, similar patterns were observed for three primary human tissues (Additional file 1: Figure S14B–D) except for greater proportion of alternative exons with novel splice-site junctions in brain (17.3% whereas other datasets contain < 10% novel alternative exons) and that the concordance between Gencode annotation and liver was much weaker than that of other datasets (31% concordance for mutually inclusive exons and 66% concordance for mutually exclusive exons).

### Characterization of the proteome of MCF-7 cells in light of full-length transcripts

The integration of full-length mRNA sequences with mass-spectrometry (MS) proteomics data provides a unique opportunity to investigate the multitude of protein isoforms that arise from transcripts that underwent alternative transcription initiation and mRNA processing. Specifically, the Iso-Seq data can serve as a source of predicted full-length open-reading frames (ORFs), which enables the detection of novel peptides from the MS data and assignment of peptides to specific protein isoforms. In traditional MS-based proteomics workflows that use canonical protein databases, such novel peptides would remain undetected or may be misassigned without the knowledge of candidate ORFs. From the 11,350 multi-exon genes and 7364 polyadenylated single-exon transcripts found in the MCF-7 cells, we could respectively identify 10,385 and 3591 ORFs that were in the range of 48–3087 amino acids (aa) in length (Additional file 1: Figure S15). A majority of 7814 genes that underwent alternative transcription initiation or mRNA processing were predicted to have coding capacity (97.4%). However, a smaller proportion of 3536 single-transcript genes (78.4%) and single-exon transcripts (48.8%) contained a predicted ORF.

To characterize the translated component of the MCF-7 transcriptome, especially in relation to the alternatively spliced isoforms, we analyzed a publicly available, deep-coverage MS dataset [42]. For MS searching, a customized protein search database was constructed, consisting of protein sequences derived from Gencode v19 (95,309 entries), ORFs predicted from MCF-7 PacBio sequences (47,325 entries), and a database of frequently observed contaminant proteins (115 entries) [43, 44]. The inclusion of the Gencode database allows for identification of any peptides derived from transcripts that may not have been detected in the PacBio data. The MS searching was done using the Morpheus algorithm [45], wherein all theoretical peptides resulting from an in silico tryptic digestion of protein entries (from Gencode, PacBio, or contaminants database) were matched against the raw mass spectra to identify peptides. We detected 38,628 unique peptides, passing a global false discovery rate (FDR) of 1%, that could be unambiguously associated with the Gencode (version 19) and/or PacBio-based predicted protein-coding sequences in MCF-7 cells. In 2872 cases, the identified peptide was only present in the Gencode database, whereas in 358 cases, the peptide was only found in the PacBio database. In addition, we found 2150 peptides associated with 481 single-exon transcripts.

**Fig. 4:**
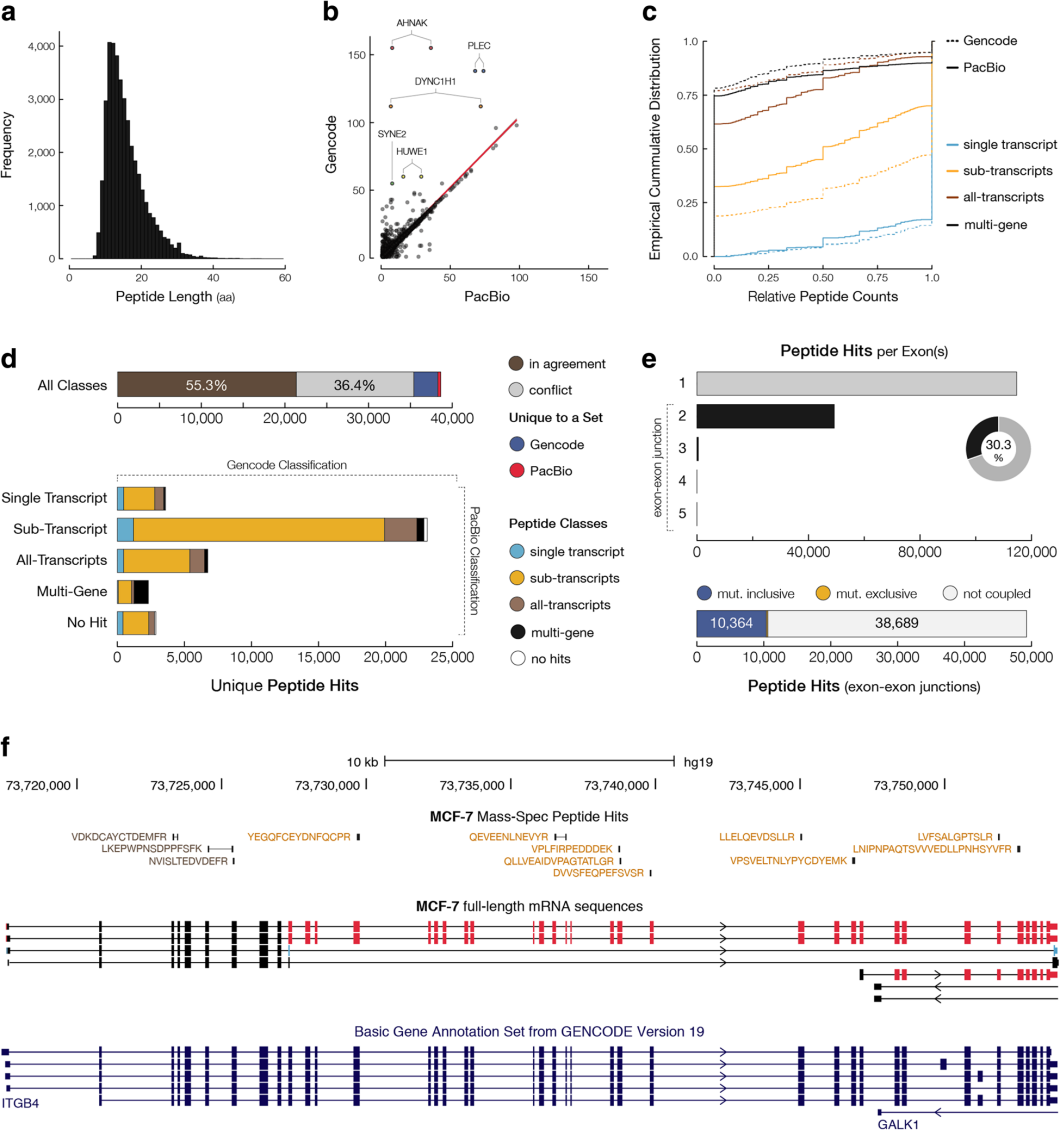
Comprehensive *map* of protein peptides supports novel alternative splicing events in full-length MCF-7 transcriptome. **a** *Histogram* shows the distribution of peptide amino acid (aa) lengths that could be associated with either Gencode or PacBio transcript variants. **b** *Scatter plot* illustrates the number of unique peptide hits per gene based on PacBio (*x*-axis) or Gencode annotation (*y*-axis). Each *dot* represents a single gene locus based on matching of PacBio and Gencode genes. **c** Empirical cumulative distribution of relative peptide counts per gene for each peptide hit category. Genes with a single transcript annotation (single-transcript category) are shown in *light blue*. Multi-transcript genes with peptides matching to a subset of transcripts (sub-transcripts category) are shown in *yellow*. Multi-transcript genes with peptides matching to all annotated transcripts (all-transcripts category) are shown in *brown*. Multi-gene hits are shown in *black*. *Dotted lines* represent the cumulative distributions based on the Gencode annotation. **d** *Bar charts* illustrate the comparison of Gencode- or PacBio-based classification of Peptides. **e** *Bar charts* show the number of peptides derived from exon–exon junctions of transcripts. The number of peptides that match exon–exon junction of mutually inclusive (*blue*) or exclusive (*yellow*) exons. **f** Peptides with different classification matching to multiple transcripts of *ITGB4*. Black peptides are all-transcripts hits whereas, based on full-length MCF-7 transcriptome data, yellow peptides are only associated with a subset of transcripts. Exons are colored based on coupling networks, shown in *red* and *blue*

Identified peptides are in the range of 7–56 aa with an average of > 15 aa in size (Fig. 4a). We observed a strong correlation (*r* = 0.96; *p* < 2.2e-16) for the number of peptides per gene based on full-length PacBio or Gencode transcripts (Fig. 4b), suggesting that full-length RNA-seq data can capture a comparable repertoire of protein-coding sequences in MCF-7 cells. Still, it is evident that for a few select ultra-long transcripts, such as *AHNAK*, *DYNC1H1*, and *PLEC*, or for particularly low abundant transcripts such as *HUWE1*, peptides were underrepresented in the PacBio database compared to Gencode (Additional file 1: Figure S16–19). It is challenging to synthesize ultra-long transcripts using current full-length cDNA synthesis protocols. For example, the central domain of *AHNAK* consists of a tandem 128 aa repeats that is absent in its ultra-long form in PacBio data, whereas the short forms were detected (Additional file 1: Figure S16). In the case that ultra-long or low-abundant transcripts are of interest for study, alternative experimental protocols are needed to enrich for such products.

The MCF-7 dataset described in this manuscript represents an in-depth characterization of a unique personal transcriptome that gives rise to unique protein sequences (i.e. different from canonical proteome reference sequences). To find evidence for potentially novel protein isoforms derived from alternatively spliced transcripts, we characterized the specificity of peptides in terms of its ability to distinguish between one or more isoforms – many peptides map ambiguously to multiple isoforms whereas others that arise from unique alternative mRNA processing events may only map to a subset of isoforms. Accordingly, peptide hits were classified into four groups, ranging from most to least specific in their mapping precision: single-transcript hits representing peptides that could uniquely map to a single isoform sequence; sub-transcript hits representing peptides that are associated with only a subset of transcript isoforms for a given gene; all-transcripts hits representing peptides that are associated with all transcripts of a given gene; and multi-gene hits that represent peptides associated with transcripts of multiple genes. This classification serves as a measure of specificity for peptide matches (Additional file 1: Figure S20) as well as evaluating the specificity of transcript annotation in representing alternatively spliced isoforms in MCF-7 cells.

Comparison between Gencode- and PacBio-based classification of peptides revealed that PacBio-based analysis of protein peptides provides a more specific peptide assignment. For example, PacBio-mapped peptides were more often associated with a subset of transcripts rather than ambiguously assigned to all transcripts. This increased specificity is due to the use of a sample-specific set of predicted protein isoforms from the MCF-7 full-length transcriptome over the Gencode annotation (Fig. 4c, d; Additional file 1: Table S3). Indeed, Gencode represents protein annotations for the entire human proteome, whereas the MCF-7 derived proteome is specific to MCF-7 cells. As shown in the empirical cumulative distribution of relative peptide counts per gene (Fig. 4c), there is a clear enrichment of peptides that discriminate between different isoforms of a gene in PacBio data vs Gencode annotation, while at the same time the overall peptide counts per gene remain the same between the two. In fact, 50% of peptides that are classified as single-gene hits (matching all transcripts of the associated gene) were classified as sub-transcript hits based on PacBio transcripts, some of which due to alternative splicing events were absent in Gencode version 19 annotation (Additional file 1: Table S3). Conversely, some peptides that were classified as single-transcript hits in Gencode were classified as sub-transcript (46%) or single-gene (18%) hits in PacBio, as many more isoforms were detected in MCF-7 cells than annotated in Gencode.

We identified 358 novel peptide hits that were missed in Gencode (Fig. 4d; Additional file 1: Figure S21). The quality of these novel peptide hits only matching PacBio-derived ORFs (“novel” peptides) were comparable to that of peptide hits matching Gencode (“known” peptides). Furthermore, peptides that had discordant classification (i.e. categorized as single-transcript in Gencode but sub-transcripts in PacBio) were also found to have a comparable quality (Additional file 1: Figure S22). The quality was assessed by statistical analysis of the distribution of peptide MS search scores (i.e. q-value or Morpheus score). We found that the discordant gene assignments for a set of peptides was primarily due to them containing single amino acid substitutions (SAS) that are present in MCF-7 cells but not in the reference sequence (Additional file 1: Figure S23). Without knowledge of SAS provided by sample-specific protein database, traditional proteomics workflows can either lead to mismatch or failure to detect peptides using general annotations such as Gencode [46]. This is more prominent for sample-specific differential expression of paralogous genes (Additional file 1: Figures S24 and S25). These observations are partly reflected by 41% multi-gene peptides that can be specifically assigned to a single gene using PacBio data.

Although most peptide hits were found within a single exon boundary, 30% were associated with exon–exon junctions covering up to five consecutive exons (Fig. 4e). From 49,263 peptides derived from parts of the transcripts that span exon–exon junctions, 10,364 peptides were associated with exons that were found to be mutually inclusive as we rarely (< 2%) observed peptides that matched mutually exclusive exons (Fig. 4e). As shown for *ITGB4* gene (Fig. 4f), differential splicing pattern in MCF-7 transcriptome can strongly influence the characterization of matching peptides given the number of isoform-specific peptides found based on the detection of novel splice isoforms that are absent in Gencode.

## Discussion

Short-read RNA-seq has become central in assessing global RNA expression patterns. However, as a result of the complexity of human transcriptome and limited size of the sequenced RNA fragments, this approach disappoints in precise reconstruction and reliable expression estimation of transcript variants [6, 7, 47]. In contrast, single-molecule long-read sequencing provides a unique opportunity to reveal the true complexity of the transcriptome [9, 10, 16, 48] as it can determine the full structure of individual transcripts by full-length sequencing.

Here, we have analyzed the deepest and longest transcriptome data so far to better understand the extent of interdependencies between transcription initiation and mRNA processing. Notably, full-length mRNA sequencing and de novo identification of high-quality (HQ) sequence of transcript variants uncovered an unprecedented amount of potentially novel transcripts in MCF-7 cells and three human tissues. Our findings not only reveal a higher level of alternative transcription initiation, splicing, and polyadenylation in MCF-7 transcriptome than previously appreciated, but also provide valuable information on the preferential selection and interdependency between these processes.

We showed that transcription initiation, splicing, and 3′ end formation are tightly coupled in > 60% of genes with multiple transcripts and such interdependencies can be found across the entire length of the mRNA molecules. Notably, we report an unforeseen and unprecedented number of genes that undergo a vigorous preferential selection during transcription initiation and mRNA processing as the choice of transcription start site subsequently influences both alternative splicing of exons and the usage of alternative poly(A) site. Ample evidence points at the critical role for RNA Pol II in the coordination between these processes (reviewed in [5, 49–51]). It has been shown that RNA Pol II initiation, pausing, and elongation rate can influence alternative splicing and polyadenylation of transcripts [52–55]. Moreover, the C-terminal domain of RNA Pol II likely acts as a scaffold for regulatory factors that are involved in splicing and polyadenylation (reviewed in [51]). Concordantly, we found an enrichment of coupling events in larger genes that seem to undergo a more extensive regulation during mRNA synthesis. However, the exact mechanisms by which the coordination is achieved remain largely unclear.

From previous studies, it became clear that polyadenylation couples with splicing machinery to influence the removal or inclusion of the last intron [18, 56, 57]. We now show that: (1) the interdependencies between splicing and polyadenylation are not necessarily restricted to the final introns; (2) that they can also involve introns that are far from the poly(A) site; and (3) that the coupling between splicing and alternative polyadenylation is not restricted to tandem 3’ UTRs. The exact mechanisms by which these coupling events are achieved fall beyond the scope of this study. Previously, it has been shown that spliceosome components are also part of the human pre-mRNA 3′-end processing complex [58]. Moreover, it is likely that there are RNA-binding proteins with a dual role in alternative splicing and polyadenylation to coordinate mRNA processing events. hnRNP H [20], CstF64 [57], MBNL1, and ELAV1 (HuR) [23, 59–61] are a few examples of such proteins. Importantly, sequences upstream and downstream of splice sites (R domains) of coupled exons were enriched for motifs that can be recognized by RNA-binding proteins with known role in regulating alternative splicing. In addition, we found MBNL binding motifs enriched in the sequences upstream of polyadenylation sites coupled with alternatively spliced exons. Interestingly, these regions lacked canonical or non-canonical poly(A) signals. This suggests that MBNL proteins mark alternative poly(A) sites and play a dual and possibly coordinating role in alternative splicing of exons and polyadenylation. This is in line with previous studies in MBNL1-deficient cells where both splicing and polyadenylation were shown to be disrupted [23, 24]. However, it is not clear to what extent these findings are biologically meaningful and if they can be extrapolated to other cell lines and cell types. Our analysis also identified a few more candidates with dual roles in mRNA processing, notably multiple RBM proteins SAMD4A, NOVA2, FUS, and SRSF2, which warrant further investigations by performing additional functional assays.

In MCF-7 cells, the multitude of protein isoforms arising from alternative transcription initiation and mRNA processing is not fully reflected in Gencode protein annotation as sample-specific set of predicted ORFs seem to provide a better specificity in discriminating peptides based on differences in ORFs derived from the same gene locus. Furthermore, as shown in this study, the presence of sample-specific single amino acid substitutions and splice-site junctions can lead to loss or mismatch of peptides when using a canonical proteome reference database. Thus, a personalized full-length transcriptome and its associated full-length ORF predictions serve as a valuable resource to capture the true complexity of the proteome and to study the global functional divergence between protein isoforms. However, it is important to note that the comprehensiveness of such databases vastly depends on the sequencing depth and library preparation strategy and, therefore, it is currently indispensable that such analyses need to be performed using the combination of Gencode and sample-specific protein sequences.

## Conclusions

This study demonstrates that our understanding of transcript structures and coordinating mechanisms that regulate transcription initiation and mRNA processing is far from complete, even in well-characterized human cell lines such as MCF-7. Single-molecule full-length RNA-seq of other human tissues also provide an additional evidence for the true complexity of the human transcriptome. Moreover, although it has been shown that single-nucleotide variants can alter the inclusion of exons in transcripts [9], it is of interest to identify variants that can affect allele-specific coupling between transcription initiation and mRNA processing. Together, these can offer a better understanding of the mechanisms that control gene regulation. As alternative splicing is a key mechanism in functional divergence of human genes, access to full-length sequence of potential protein isoforms allows us to better understand biological function through examining interactions and cross-tissue dynamics of protein isoforms [62]. In turn, this unique set of protein isoform interactions serves as a global view of protein functional repertoire and thereby provide valuable insights into underlying mechanisms of diverging physiological, developmental, or pathological conditions.

## Methods

### RNA sample preparation, library preparation, and sequencing

Total RNA of the MCF-7 cell line was purchased from Biochain. The cDNA library was constructed using the Clontech SMARTer cDNA kit. The majority of the size selection and sequencing was performed using agarose gel cutting at 1–2 kbp, 2–3 kbp, and > 3 kbp. A total of 119 SMRT cells (1–2 kbp for 37 cells, 2–3 kbp for 37 cells, > 3 kbp for 33 cells, and 12 cells for no size selected library) were sequenced for the first set of libraries using P4-C3 chemistry and 2-h movies. Later, with improved chemistry and size selection method, additional libraries were made using the same Clontech SMARTer cDNA kit followed by size selection using SageELF to create fractions at 0–1 kbp, 1–2 kbp, 2–3 kbp, 3–5 kbp, and 5–7 kbp. Sequencing was done using P5-C3 chemistry and 4-h movie time for a total of 28 SMRT cells (0–1 kbp for 4 cells, 1–2 kbp for 5 cells, 2–3 kbp for 5 cells, 3–5 kbp for 7 cells, 5–7 kbp for 7 cells). Together, 147 SMRT cells were sequenced. The schematic overview of the library preparation, sequencing, and data analysis is provided in Additional file 1: Figure S26.

The methodologies and experimental settings for RNA preparation, cDNA synthesis, library preparation, and sequencing are further described at https://github.com/PacificBiosciences/DevNet/wiki/IsoSeq-Human-MCF7-Transcriptome. We downloaded the 2015 dataset, which is an updated version of the original 2013 release. In addition, we used publicly available data from three human tissues (brain, heart, and liver) for comparative analysis. These datasets are described at http://www.pacb.com/blog/data-release-whole-human-transcriptome/.

### Annotation of full-length high-quality transcripts using isoform-level clustering algorithm

The identification, polishing, and annotation of transcripts were previously carried out using the Iso-Seq bioinformatics pipeline made public by Pacific Biosciences. A full description of the algorithm is available in a previous publication [15]. Reads were first classified into full-length and non-full-length based on the presence of 5′ and 3’ cDNA primers, as well as the polyA tail preceding the 3′ primer. To find transcript clusters, an isoform-level clustering algorithm (ICE) performs a pairwise alignment and reiterative assignment of full-length reads to clusters based on likelihood. After ICE, partial reads are added to the isoform clusters to increase coverage for a final consensus using the Quiver algorithm [63]. The output from the bioinformatics pipeline is a set of full-length transcript sequences that can be mapped to the reference genome for identifying genes and isoform relationships. For further information on the methodology and experimental settings visit https://github.com/PacificBiosciences/IsoSeq_SA3nUP/wiki.

Based on the Quiver algorithm’s predicted consensus accuracy, transcript sequences that had a predicted accuracy of > 99% (excluding QVs from the first 100 bp and last 30 bp due to occasionally insufficient coverage for accurate estimation of accuracies) were considered HQ transcripts and used for further analysis. The HQ transcript sequences were mapped back to the human genome (hg19) and filtered for > 99% alignment coverage and > 85% alignment identity. Of 280,051 HQ transcripts in MCF-7, nine did not map to hg19, 13,543 were filtered due to low coverage, and 14 were filtered due to low identity. Redundant transcripts were then collapsed to create a final dataset used in this study.

### Comparison to the GENCODE annotation

We used GENCODE annotated transcripts (version 19) as reference to compare with the identified transcripts in the human MCF-7 transcriptome data. The comparison for transcript annotations was carried out using *cuffcompare* from the Cufflinks suite [64].

To identify interdependent alternative exons that are already represented in the Gencode annotation, for each exon–exon coupling, we performed the following: (1) all transcripts that encompass both interdependent exons; (2) assessed whether the same splice-site junctions were annotated; (3) counted the number of transcripts that contain both exons, only one of the two exons, or none; (4) relative counts were calculated based on the total number of transcripts that encompassed the two exons. For each dataset, the relative counts of co-occurrence and independent observations were compared for mutually inclusive and mutually exclusive exons separately.

### Comparison to standard RNA-seq datasets

We used five publicly available RNA-seq datasets (SRR1035698, SRR1107833, SRR1107834, SRR1107835, and SRR1313067; generated on Illumina HiSeq2000 or Illumina HiSeq2500 platforms) to evaluate the reliability of gene expression quantification based on full-length mRNA sequencing data used in this study. As accurate transcription reconstruction is not feasible for short-read RNA-seq data, the comparison is made at the gene level using GENCODE annotation (version 19). As part of the GENTRAP pipeline [65], GSNAP aligner with default parameters was used to align paired-end Illumina reads to the human genome (hg19). Next, HTSeq [66] with default parameters was used to calculate the fragment counts per gene, which was subsequently adjusted for gene length to provide a median measure of gene expression. All statistical analyses were performed in R.

### Definition of transcription start site, polyadenylation site, and donor and acceptor splice sites

In this study, by processing the GFF file that contains the annotation of all identified transcripts and exon/intron boundaries (defined by the genomic position and strand on the hg19 reference sequence), a list of all transcription and mRNA processing events is produced. TSSs are defined as the first genomic position of each transcript structure. PASs are defined as the last genomic position of each transcript. The most upstream and downstream genomic positions of exons were used to define donor and acceptor splice sites, respectively. However, for the first exon only the donor site is described as the first position is defined as transcription start site. Likewise, the last exon does not contain a donor splice site as the position is defined as polyadenylation site. If multiple transcripts share the same feature, then only one copy is kept in the unique set of features at each locus. Furthermore, the union of all unique exons is defined as the available sequence at each locus. This is also illustrated in Fig. 1b. Terminal positions of transcripts were curated based on a 10-bp window to remove stochastic noise and minimize the number of false TSS and PAS for each locus.

### Comparison to Encode CAGE and RNA-PET datasets

We used the publicly available MCF-7 cells CAGE (GSM849364) and RNA-PET (GSM1006905) datasets from the Encode project to evaluate the reliability of 5′ and 3′-ends of identified transcripts in PacBio data. We used Bedtools to identify base-pair distance between CAGE tags and TSSs found in PacBio data. For RNA-PET, both 5′ and 3′-ends were simultaneously compared to TSSs and PASs identified in PacBio to report the distances for the best matching full-length transcripts. All data processing was done using Bedtools closest function. Since Bedtools may report multiple hits, we have selected the one with the least number of differing nucleotide positions as the best hit.

### Alignment and quantification of supporting reads for each transcript

The number of reads aligned to each transcript was used as the supporting evidence for each transcript structure. To identify the number of supporting reads, the polished sequences of all unique transcripts were used as a reference for the unique alignment of raw reads using BLASR [67]. Other parameters were set to default and according to the Pacific Biosciences guidelines.

### Statistical analysis

After defining unique features (TSSs, exons, and PASs) and identifying the number of supporting reads for transcripts at each locus, all possible pairwise comparisons between features were made. To do this, a two-by-two contingency table is constructed based on the following criteria: (1) two features should not partially or fully overlap; (2) dependency between alternative TSSs and features that are located in their upstream region are omitted as their exclusivity is given. The same rule is applied to downstream of alternative PASs; (3) only transcripts that fully encompass the region that is represented by two features are used to populate the two-by-two contingency table. This is to ensure that all counts are based on direct observation of their mutual inclusion/exclusion in identified transcripts; (4) single-exon transcripts are excluded from the analysis; (5) only multi-transcript loci are examined for possible interdependencies between alternative transcript initiation, alternative splicing, and alternative polyadenylation. The table describes the number of times two features are observed in the same transcript or exclusively, as well as the sum of reads that are mapped to transcripts that do not support the presence of either features (Fig. 1c). A significant coupling between two features is assessed using Fisher’s exact test. The mutual inclusivity or exclusivity of coupled features are defined using their log-transformed odds ratio. All *p* values are adjusted using Bonferroni multiple testing correction.

Coupling network is constructed based on detected interdependencies between pairs of features for each gene. Nodes represent features and mutual inclusivity or exclusivity is represented by black or red edges, respectively, in the network. Mutual inclusivity or exclusivity sub-networks are constructed after removing all the other edges. No further filtration is performed on gene coupling networks.

### Sanger sequencing validation

The polymerase chain reaction (PCR) for Sanger sequence validation was performed using the 2× Phusion High-Fidelity PCR master mix with HF buffer (NEB). Briefly, the PCR ran for 30 cycles with 1-min elongation at 72 °C. The PCR products were purified using Ampure XP beads following the guidelines of the manufacturer. The sizing of the amplicons was checked using Agilent’s Labonachip system. The Sanger sequencing of the products was performed by the LGTC and the sequences were analyzed using Sequence Scanner Sofware 2 (Applied Biosystems, CA USA).

### Single molecule RNA fluorescence in situ hybridization

Single-molecule RNA FISH relies on the combined fluorescence from 25 to 48 singly fluorophore labeled oligonucleotides bound to the same RNA. By using the fluorescence from a guide probe set in one dye, the fluorescence from one or more exon-specific probe sets with < 25 oligonucleotides, and each labeled with a separate dye, can be accurately registered as belong to the same RNA. The optimal number of oligonucleotides per specific set must be experimentally determined.

#### Probe sets

Four probe sets were designed at www.biosearchtech.com/stellarisdesigner to detect: (1) the common exons of the human CALU (Calumenin; NCBI Gene ID: 813; 7q32.1) mRNAs (CALU_E); (2) the alternatively spliced exon 4 (CALU_E4); (3) the alternatively spliced exon 5 (CALU_E5); (4) and the common first intron (CALU_I1). The probe set target sequences were as follows: CALU_E: NM_001199671.1 nts 1–141, 957–1188, 1383–1610, 1811–2875; CALU_E4: NM_001199671.1, nts 1177–1394; CALU_E5: NM_001199672.1, nts 1177–1394; and CALU_I1: NC_000007.14, nts 128,739,433–128,747,432. CALU_E is an inclusive probe set designed to detect the following variants: NM_001219.4, NM_001130674.2, NM_001199671.1, NM_001199672.1, NM_001199673.1, and NR_074086.1. Both CALU_E and CALU_I1 are full sets with > 32 oligonucleotides, whereas the sets targeting the short exons 4 and 5 have nine oligonucleotides each. The four sets were synthesized at LGC Biosearch Technologies as custom Stellaris® probe sets with unique fluorophores: CALU_E: Quasar® 670; CALU_E4: Quasar 570; CALU_E5: Cal Fluor ® Red 610; and CALU_I1: FAM. The CALU_E4 and CALU_E5 probes were further purified by reverse phase HPLC, to ensure full labeling.

#### Reagents and smRNA FISH

Human breast adenocarcinoma MCF-7 cells (ATCC-HTB-22) were obtained from ATCC (Manassas, VA, USA) and cultured as recommended by the provider. The hypotriploid karyotype is available at the provider’s website and shows three chromosomal loci for 7q32.1. 2-(4-Amidinophenyl)-6-indolecarbamidine dihydrochloride, 4′ 6-Diamidino-2-phenylindole dihydrochloride (DAPI), molecular biology grade ethanol, acetic acid, and methanol were from Sigma Aldrich (St. Louis, MO, USA). Vectashield was from Vector Laboratories (Burlingame, CA, USA). Stellaris RNA FISH hybridization and wash buffers were from LGC Biosearch Technologies. Stellaris RNA FISH was performed as previously described for methanol/acetic acid-fixed cultured cells [68, 69].

#### Image acquisition and analysis

DAPI-stained nuclei, fluorescein (FAM), Quasar 570 (Q570), CalFluor 610 (CF610), and Quasar 670 (Q670) dyes were imaged through a 60X 1.4NA oil-immersion lens on a Nikon TI widefield microscope using the appropriate filters: 49000-ET-DAPI; 49011-ET-FITC; SP102v1; SP103v2; 49022-ET-Cy5.5, respectively. The exposure and sequence of channels to acquire were determined based on the brightness and photostability of the dye with which each probe set was labeled. The sequence of exposure was Q670, followed by FAM, and then either Q570 or CF610, or both. Each Z-slice was exposed for 1 s, except for Q670 which required 2-s exposures. For each field of view, a range spanning the vertical dimension of the cell (typically 10 um) is defined and for each channel, a series of images were acquired through this span at 0.3 μm increments by using Nikon Elements’ Advanced Research software.

Each Z-series was collapsed and rendered as a single, max-intensity projected image. Translational registration to align images shifted relative to another was accomplished by ImageJ macros after identification of a region containing overlapping signals in each channel. Peak positions of these signals were determined relative to each other to inform the shift of each channel. Next, spots and their centroid positions were identified in each channel using the ImageJ Find Maxima utility. These positions were then compared to one another and co-localized spots were grouped if within 3 pixels (330 nm). Based on these groupings, spots were categorized into separate transcript variants and displayed on the image for review. Finally, cell borders were defined and spots associated with distinct cells for per-cell and per-transcript variant copy number determination. RNA FISH features were counted in at least ten cells.

### Sequence motif analysis relative to polyadenylation sites

For each detected locus, we reported the last nucleotide as polyadenylation site. Each genomic location was converted into a BED format. Strand-specific genomic sequences located up to 35 nt upstream of each unique polyadenylation site were extracted, in a FASTA format, using UCSC Table Browser (GRCh37/hg19). FASTA files were parsed using a custom bash script to count the number of sequences containing specific 6-mer motifs: one of the two canonical polyadenylation signals AATAAA and ATTAAA; or one of the 11 non-canonical polyadenylation signals (AAGAAA, AATACA, AATAGA, AATATA, AATGAA, ACTAAA, AGTAAA, CATAAA, GATAAA, TATAAA, TTTAAA). Subsequently, the same 6-mer motifs were counted for each unique PAS significantly coupled to TSSs or exons and for each unique PAS that did not show a significant coupling.

For PASs that could not be attributed to known poly(A) signals, we ran DREME [70] (v. 4.11.4) to identify enriched motifs. A randomly shuffled set of sequences was generated from the original sequences of the examined PASs and used as a background set. In addition, the sequences of known recognition motifs for MBNL proteins [24–26] were counted for each set using a custom script. Subsequently, the enrichment of each motif was assessed by Fisher’s exact test.

### Tandem 3’ UTR analysis

This analysis was performed to identify loci that contain tandem 3’ UTRs (loci that contain more multiple PASs located in the same last exon). Custom scripts were used to identify loci that contain at least two PASs that share the same coordinates of the last exon start. The number of loci with tandem 3’ UTRs was calculated for those in which PAS was significantly coupled to alternative exons and for those that did not show any significant interdependencies between alternative exons and the PAS usage.

### Sequence motif analysis relative to acceptor and donor sites

For each detected locus, we reported the first and last nucleotide of each exon as acceptor splice site and donor splice site, respectively. Each unique genomic position was converted into a BED format and the strand specific sequences of 2 nt in length were extracted using UCSC Table Browser (GRCh37/hg19) for both acceptor and donor splice sites. A custom bash script was used to count the number of dinucleotide sequences containing “GT” and/or “AG.”

### RNA-binding motif analysis

We used MEME suite tools to identify enriched sequence motifs present in exons significantly coupled with TSSs, PASs, or other alternative exons. For each unique exon, three regions were considered: R1 (containing up to 35 nt upstream of the acceptor splice site); R2 (containing 32 nt downstream of the acceptor splice site and 32 nucleotides upstream of the donor splice site), and R3 (containing up to 40 nt downstream of the donor splice site). The R1, R2, and R3 regions were obtained by extracting strand-specific FASTA sequences using UCSC Table Browser (GRCh37/hg19).

We locally ran DREME [70] (v. 4.11.4) for each region separately and performed a motif search using a negative background (R1, R2, and R3 regions from exons that were not significantly coupled). In each case, a maximum of ten motifs with E-values < 0.05 was reported. The remaining parameters were kept as default. We then compared each motif found by DREME against the human RNA-binding motifs database CISBP-RNA using TOMTOM Motif Comparison tool [71]. We ran the analysis by setting the Pearson correlation coefficient as comparison function and considered only matches with a minimum FDR (q-values) < 0.05.

### Open-reading frame prediction and proteomics data analysis

ORF prediction was done on the PacBio MCF-7 sequences using ANGEL [72]. Prediction was done on both the PacBio consensus reads and a genome-corrected version of the transcript; whichever produced the longer ORF was chosen to represent the transcript CDS. The predicted MCF-7 ORF sequences were concatenated with Gencode version 19 and protein sequences representing common mass spectrometry (MS) contaminants, creating a customized FASTA file (i.e. proteomics search database). The Morpheus software (version 131) was employed for MS searching of the custom database against the MCF-7 Thermo Raw files obtained from the study by Geiger et al. [42]. Unknown precursor charge state range was set to + 2 to + 4. Absolute and relative MS/MS intensity thresholds were disabled. Maximum number of MS/MS peaks were set to 400. Assign charge state was set to true. De-isotoping was disabled. The protease specificity was set to trypsin with no proline rule enabled. Up to 1 missed cleavage was allowed and N-terminal methionine truncations was variable. Fixed modifications used were carbamidomethylation of cysteines. Variable modification used was oxidation of methionines. Precursor mass tolerance used was 2.1 Da (monoisotopic) and product mass tolerance was 0.025 Da (monoisotopic). Modified forms of the same peptide were collapsed and treated as one peptide identification for calculation of FDR. An FDR of 1% was used to filter for final peptide identifications. All identified peptides were categorized as: single-transcript if the peptide matches to only one gene with one transcript; sub-gene if the peptide matches to a subset of transcripts of only one gene; single-gene if the peptide matches to all transcripts of only one gene; and multi-gene if the peptide matches to multiple transcripts from multiple genes.

### Data availability

Iso-seq datasets used in this manuscript were provided by Pacific Biosciences and are publicly available [73–75]. For the comparison with standard RNA-seq, the following publicly available RNA-seq datasets were used: SRR1035698, SRR1107833, SRR1107834, SRR1107835, and SRR1313067. To assess the reliable detection of 5′- and 3′-ends of identified transcripts, we used the publicly available MCF-7 cells CAGE (GSM849364) and RNA-PET (GSM1006905) datasets from the Encode project. All Python scripts and research data are made open-source and publicly available at *Zenodo* public repository [76]. In addition, a detailed description of the methodologies along with all open-source Python scripts and generated results are also made publicly available at https://git.lumc.nl/s.y.anvar/mRNA-Coupling/wikis/home.

## Additional files

Additional file 1: This document contains additional supporting evidence for this study that are presented in form of supplemental tables and figures. (PDF 11005 kb)

Additional file 2: Reviewer reports and authors’ response to reviewers. (DOCX 30 kb)
