# Supplementary material for: Full-length mRNA sequencing uncovers a widespread coupling between transcription initiation and mRNA processing
Source: Genome Biol. 2018 Mar 29;19:46. doi: 10.1186/s13059-018-1418-0 (PMC5877393; doi:10.1186/s13059-018-1418-0)
Supplement: Supplementary file 1 — This document contains additional supporting evidence for this study that are presented in form of supplemental tables and figures. (PDF 11005 kb) [file 13059_2018_1418_MOESM1_ESM.pdf]

Additional file 1

Full-length mRNA sequencing uncovers a widespread coupling between transcription initiation and mRNA processing

Seyed Yahya Anvar[1,2,3,*], Guy Allard[1], Elizabeth Tseng[4], Gloria Sheynkman[5,6], Eleonora de Klerk[1,7], Martijn Vermaat[1,2], Raymund H. Yin[8], Hans E. Johansson[8], Yavuz Ariyurek[1,2], Johan T. den Dunnen[1,2], Stephen W. Turner[4], and Peter A.C. 't Hoen[1]

[1] Department of Human Genetics, [2] Leiden Genome Technology Center, and [3] Department of Clinical Pharmacy and Toxicology, Leiden University Medical Center, Leiden, 2300 RC, The Netherlands. [4] Pacific Biosciences, 1305 O'Brien Drive, Menlo Park, CA 94025, USA. [5] Center for Cancer Systems Biology (CCSB) and Department of Cancer Biology, Dana-Farber Cancer Institute, Boston, MA 02215, USA. [6] Department of Genetics, Harvard Medical School, Boston, MA 02115, USA. [7] Department of Microbiology and Immunology, UCSF Diabetes Center, University of California San Francisco (UCSF), San Francisco, CA 94143-0534, USA. [8] LGC Biosearch Technologies, Petaluma, CA 94954-6904, USA.

* To whom correspondence should be addressed:

SYA Tel: 0031715268559; Email: s.y.anvar@lumc.nl

Table S1 – Overview of full-length mRNA sequencing using Pacific Biosciences RS II.

Sequencing	Size selection *	# SMRT Cells
P4-C3 chemistry	1 – 2 Kb	37
	2 – 3 Kb	37
	> 3 Kb	33
	Not size selection	12
P5-C3 chemistry	0 – 1 Kb	4
	1 – 2 Kb	5
	2 – 3 Kb	5
	3 – 5 Kb	7
	5 – 7 Kb	7
	Total	147

** At this point, size selection is needed to balance the change of preferential loading of molecules in ZMWs. Thus, relatively narrow size ranges were used during size selection. However, to minimize potential boarder effects during size selection, the sequencing libraries do have some overlap in the size of molecules that are present. In addition, 12 SMRT cells that were used for libraries without any size selection further aids in removing potential impacts of size selection procedure. Distribution of reads that capture the entire mRNA molecule reflects the expected distribution of fragments present in the library.*

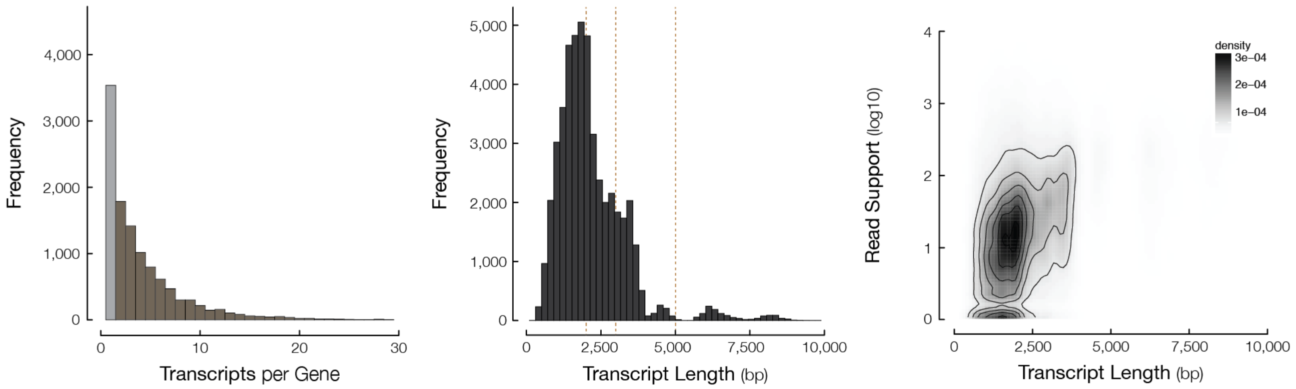

Figure S1 – Overview of identified transcripts in MCF-7 transcriptome. Histograms show the distribution of the number of identified transcripts per gene and transcript lengths. Density plot depicts the number of supporting reads based on transcript length. The number of supporting reads does not correlate with the length of full-length transcripts.

Table S2 – The comparison of detected transcript structures with GENCODE v.19 annotation. The total of 44,531 transcript variants that are detected in the MCF-7 human breast cancer transcriptome have been assessed.

Class	# Transcripts	% Transcripts
Complete match of intron chain	13,955	28.9%
Detected transcript is contained in one of GENCODE annotated transcripts	9,255	19.1%
Potentially novel transcript variant (at least one splice junction is shared)	23,871	49.3%
Single exon overlapping a reference exon and at least 10bp of an intron	0	0.0%
Transcript structures that entirely fall within a reference intron	49	0.1%
Generic exonic overlap with a reference transcript	902	1.9%
Possible polymerase run-on fragment (within 2kb of a reference transcript)	0	0.0%
Repeats, determined by looking at the soft-masked reference sequence	0	0.0%
Unknown intergenic transcripts	195	0.4%
Exonic overlap with reference on the opposite strand	94	0.2%
An intron of the detected transcript overlaps with an intron from the opposite strand	69	0.1%

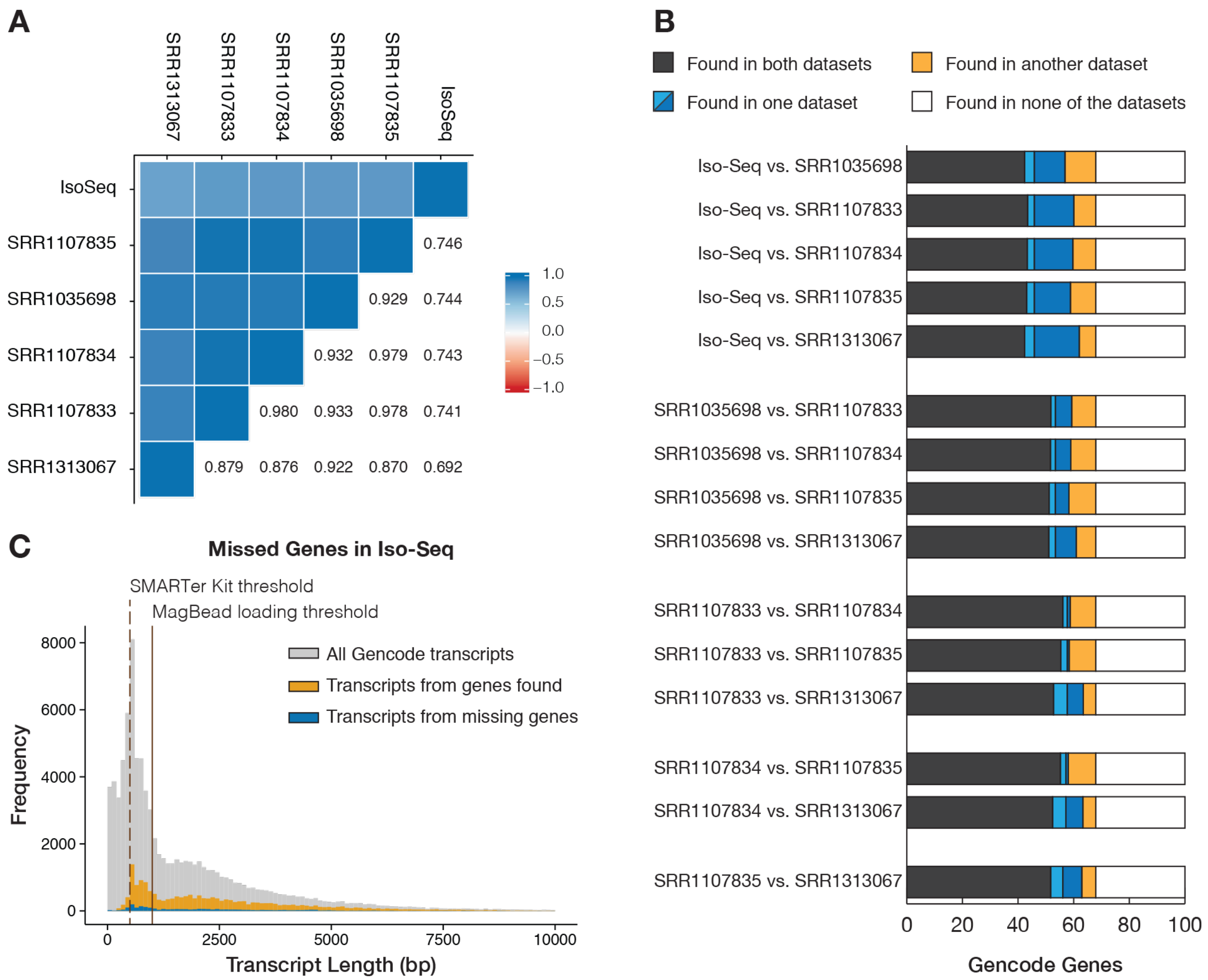

Figure S2 – Comparison of gene detection and expression quantification between Pacific Biosciences RSII and Illumina HiSeq2000 platforms. **A)** Heat map shows inter-dataset Spearman correlation of quantified gene expression. The GENCODE version 19 was used for annotation as short-read sequencing data could not be reliably used to reconstruct transcript structures. IsoSeq represents full-length mRNA sequencing data generated by Pacific Biosciences RSII platform whereas datasets with accession numbers starting with SRR are publicly available RNA-Seq datasets produced by Illumina HiSeq2000. **B)** Bar charts depict proportion of overlapping genes that are detected in different datasets. Black bars depict overlap between paired datasets. Blue bars represent proportion of genes that are detected in one of the paired datasets. Orange bars show the proportion of genes that are not detected by either dataset but have been detected in other datasets that are included in this study. Proportion of genes that do not express in MCF-7 cell lines are shown by white bars. **C)** Size of annotated transcripts for consistently detected genes that were missed in full-length mRNA sequencing data are plotted (blue) against those that are found in full-length mRNA sequencing data (orange). Grey histogram represents the distribution of the size of all transcripts that are annotated in GENCODE version 19. Dashed line depicts 500bp mark for SMARTer full-length cDNA synthesis protocol that preferentially removes short RNA molecules. Depicted by a vertical line, Magbead loading threshold that is used for optimal sequencing, limiting the number of short molecules (<1kbp) to be sequenced.

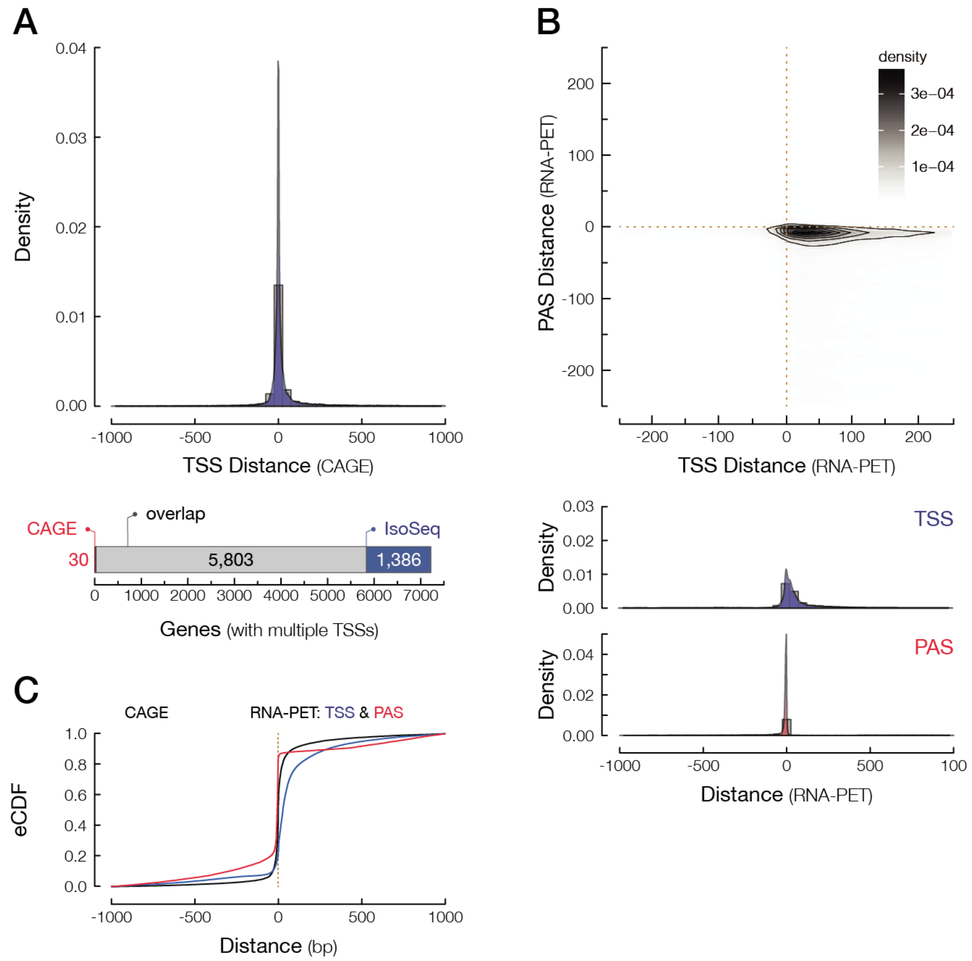

Figure S3 – Concordance between Encode CAGE and RNA-PET datasets and transcription start sites (TSSs) and polyadenylation sites (PASs) that are detected in PacBio MCF-7 dataset. **A)** Distribution of CAGE tag distance to the closest TSS in full-length MCF-7 transcriptome. Bar chart shows the number of multi-TSS genes that overlap between Encode CAGE dataset and PacBio IsoSeq. **B)** 2D density plot of distances for the TSS and PAS of the closest full-length transcript to each Encode RNA-PET 5' and 3' tag pairs. Individual distribution plots for TSSs and PASs are also provided. **C)** Empirical cumulative distribution of distances for CAGE and RNA-PETs are illustrated across the entire set.

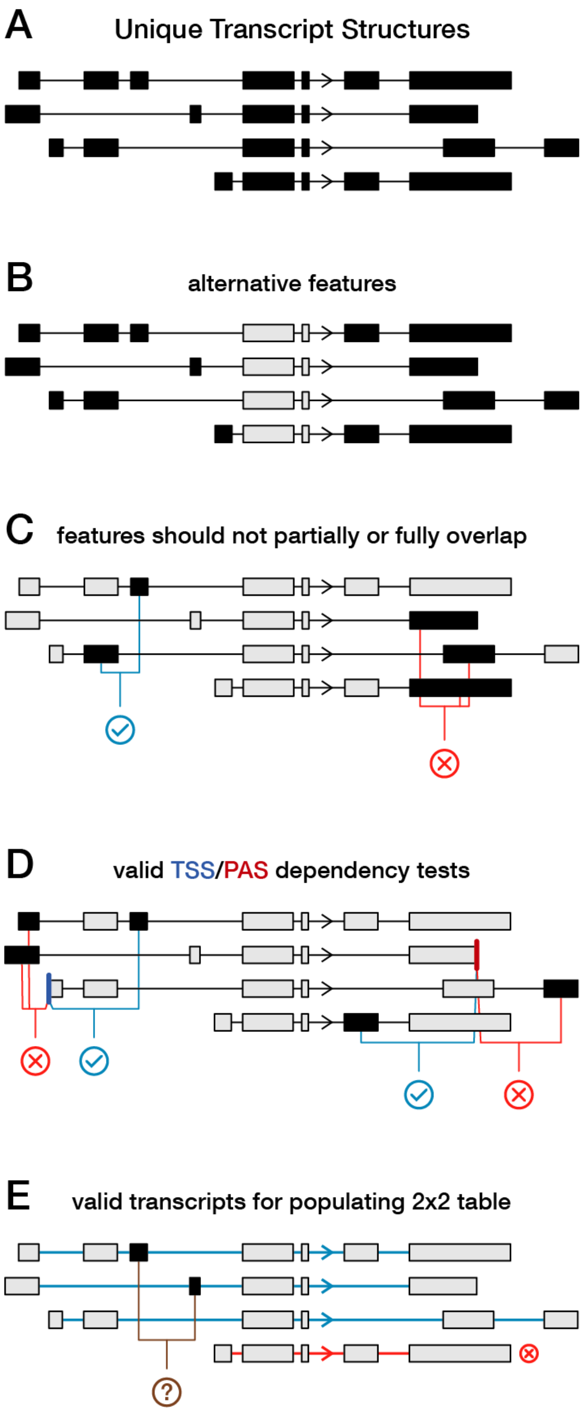

Figure S4 – The criteria for evaluating the interdependency between features representing alternative transcription initiation and mRNA processing. **A)** only multi-transcript loci are examined for possible interdependencies between alternative transcript initiation, alternative splicing and alternative polyadenylation. In addition, single-exon transcripts are excluded from the analysis. **B)** Alternative TSSs, alternative exons and alternative PASs are considered for the analysis. i.e., constitutive exons are excluded. **C)** two features should not partially or fully overlap. **D)** dependency between alternative TSSs and features that are located in their upstream region are omitted as their exclusivity is given. The same rule is applied to downstream of alternative PASs. **E)** only transcripts that fully encompass the region that is represented by two features are used to populated the two-by-two contingency table. This is to ensure that all counts are based on direct observation of their mutual inclusion/exclusion in identified transcripts.

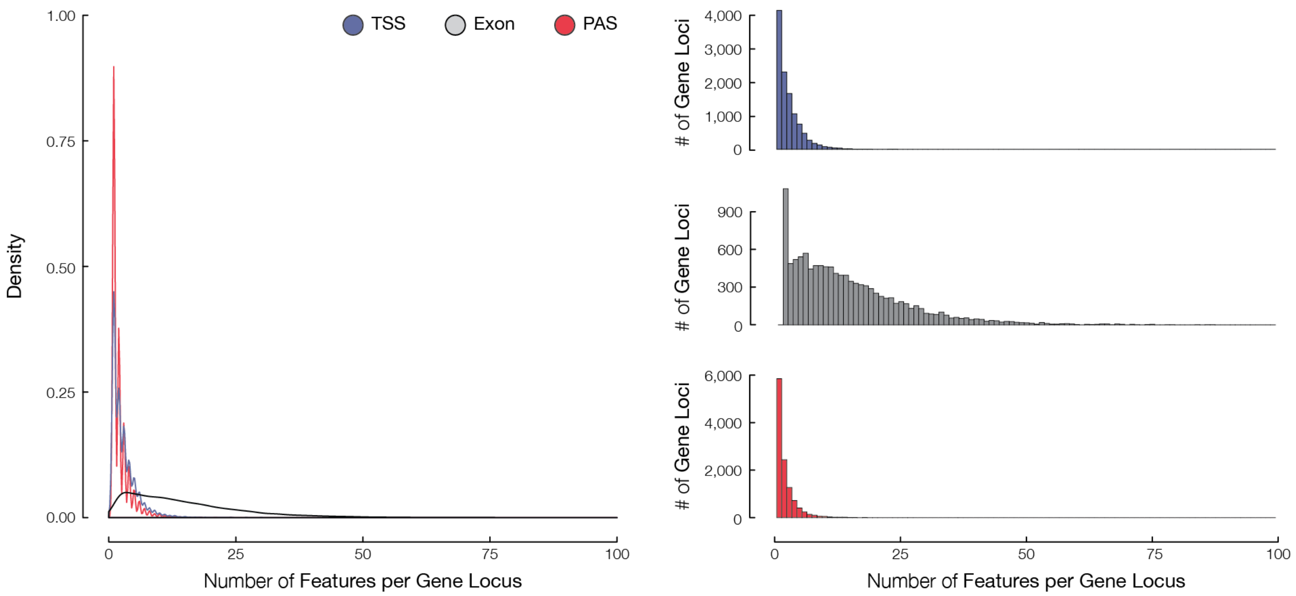

Figure S5 – The distribution of the number of transcription start sites (TSSs), exons, and polyadenylation sites (PASs) that are detected at each locus. In total, 33,437 TSSs, 168,783 exons and 24,950 PASs were identified and attributed to gene loci. The histogram distributions for the number of unique TSSs, PASs and Exons at each locus are illustrated in blue, red and grey, respectively.

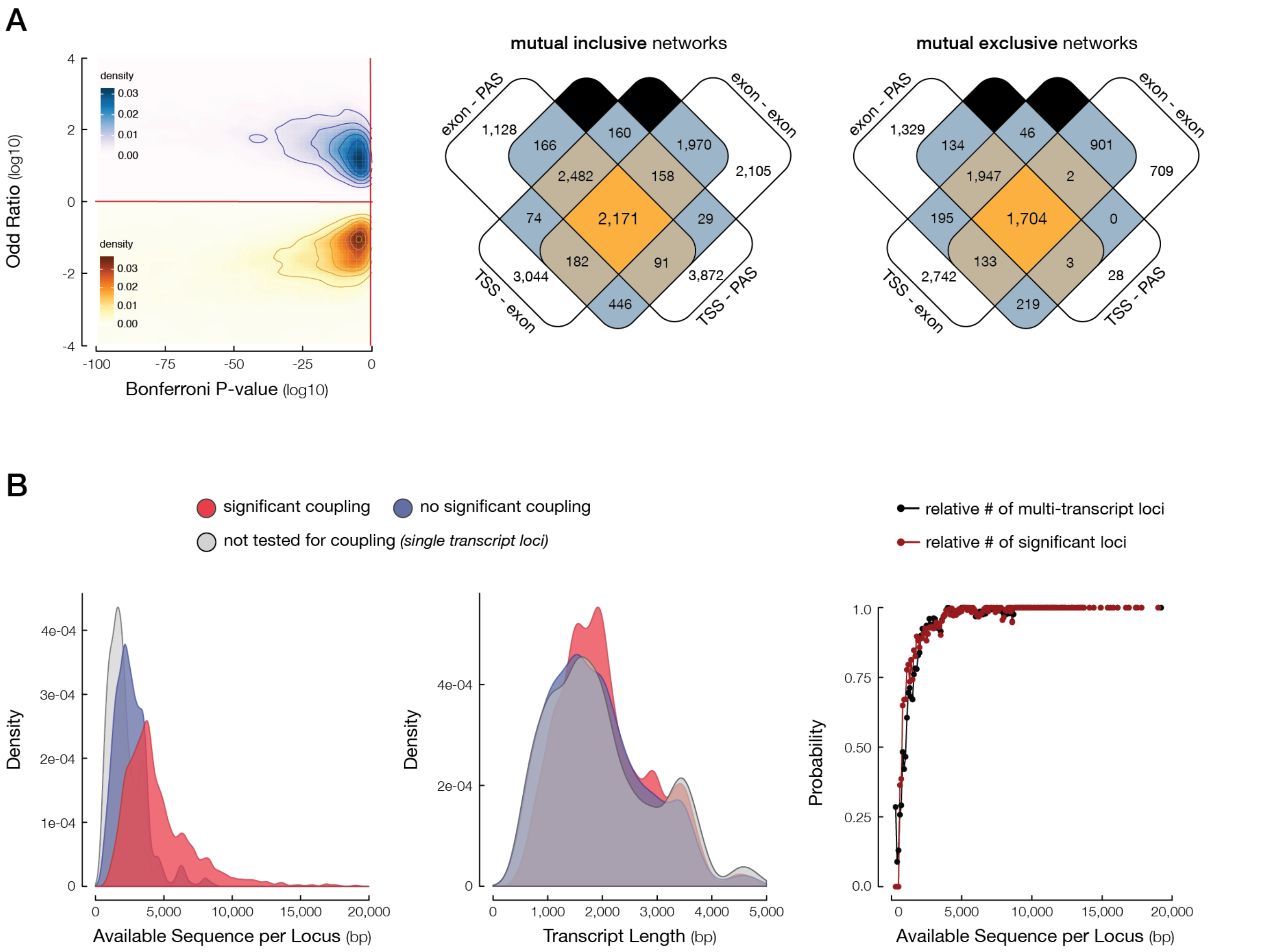

Figure S6 – The mutual inclusivity or exclusivity of feature-pairs, mostly enriched in larger gene loci but not associated with transcripts length. **A)** Density plot illustrates significantly linked features that are colored according to the type of linkage. Mutually inclusive pairs are colored in blue whereas those pairs that are mutually exclusive are illustrated in dark yellow. The vertical red line depicts the adjusted p-value threshold of 0.05 for significant coupling. Non-linked pair incidences are plotted in grey dots. Venn diagrams show the number of mutual inclusive/exclusive networks that represent different classes of links: TSS-exon, TSS-PAS, Exon-Exon and Exon-PAS. **B)** Distribution of available sequence per locus or transcript lengths per locus with only one transcript variant (not tested; plotted in grey), loci with multiple transcript variants that do not have any significantly linked feature-pairs (blue) and transcripts with significantly coupled feature-pairs (red). Line plots show the relative number of loci with multiple transcripts (black) and the relative number of multi-transcript loci with significant coupling that are plotted against the length of available sequence per gene locus. 50bp bins were used to group examined transcripts by length.

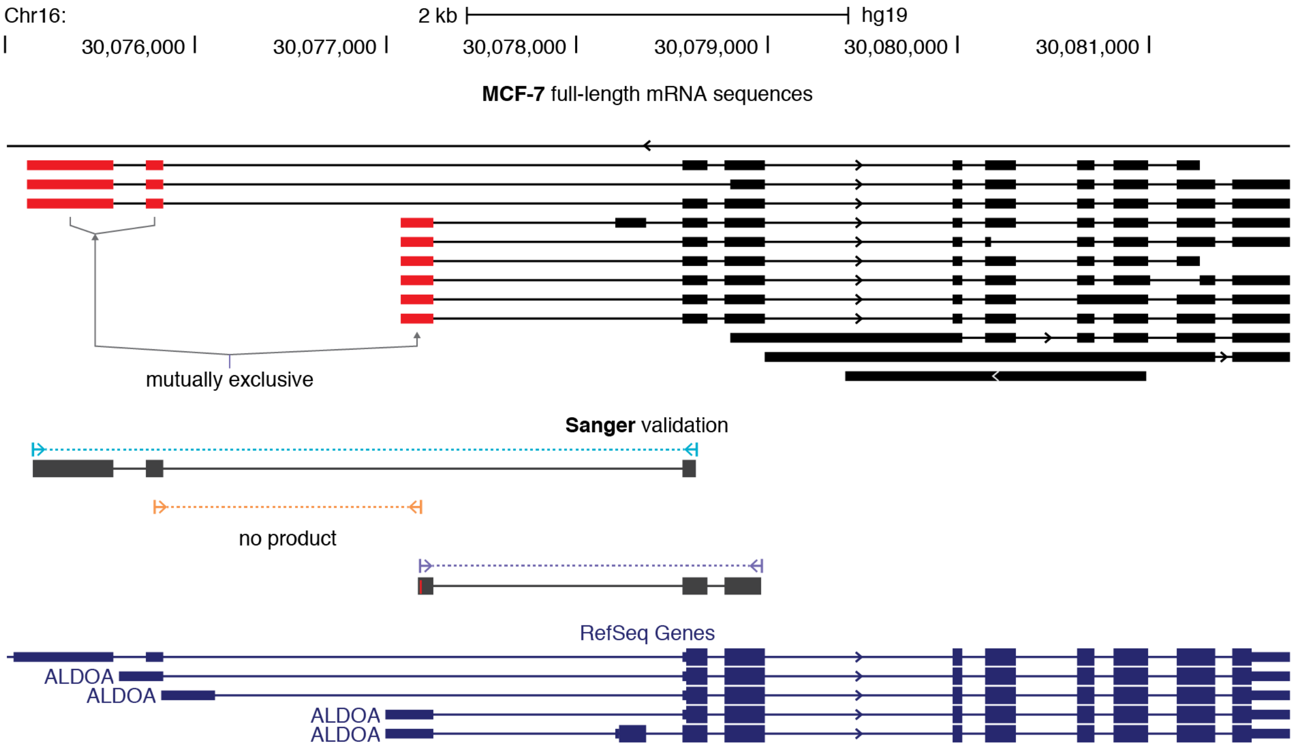

Figure S7 – The interdependence of transcription and mRNA processing of *ALDOA*. Alternative transcription start sites affect the inclusion or exclusion of alternative exons. Targeted Sanger sequencing supports the mutual exclusive events depicted in red. Mutual exclusivity of the alternative first exons are also supported in RefSeq annotation.

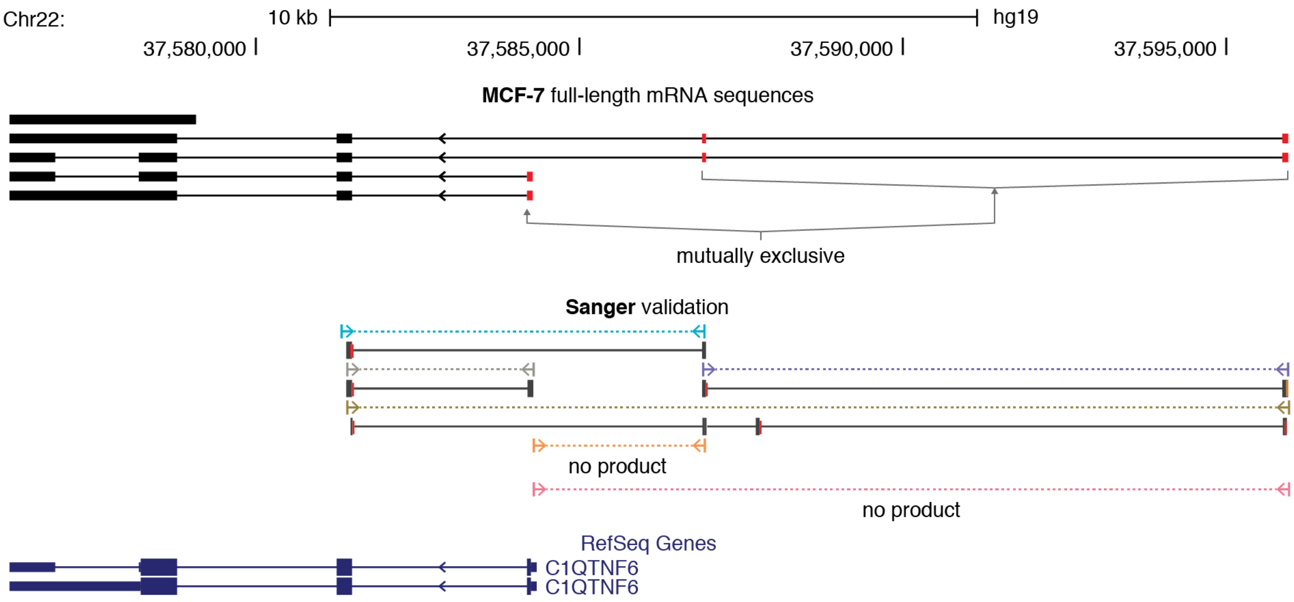

Figure S8 – The interdependence of transcription and mRNA processing of *C1QTNF6*. Alternative transcription start sites affect the inclusion or exclusion of alternative exons. Targeted Sanger sequencing supports the mutual exclusive events depicted in red. Mutual exclusivity of the alternative first exons are also supported in RefSeq annotation.

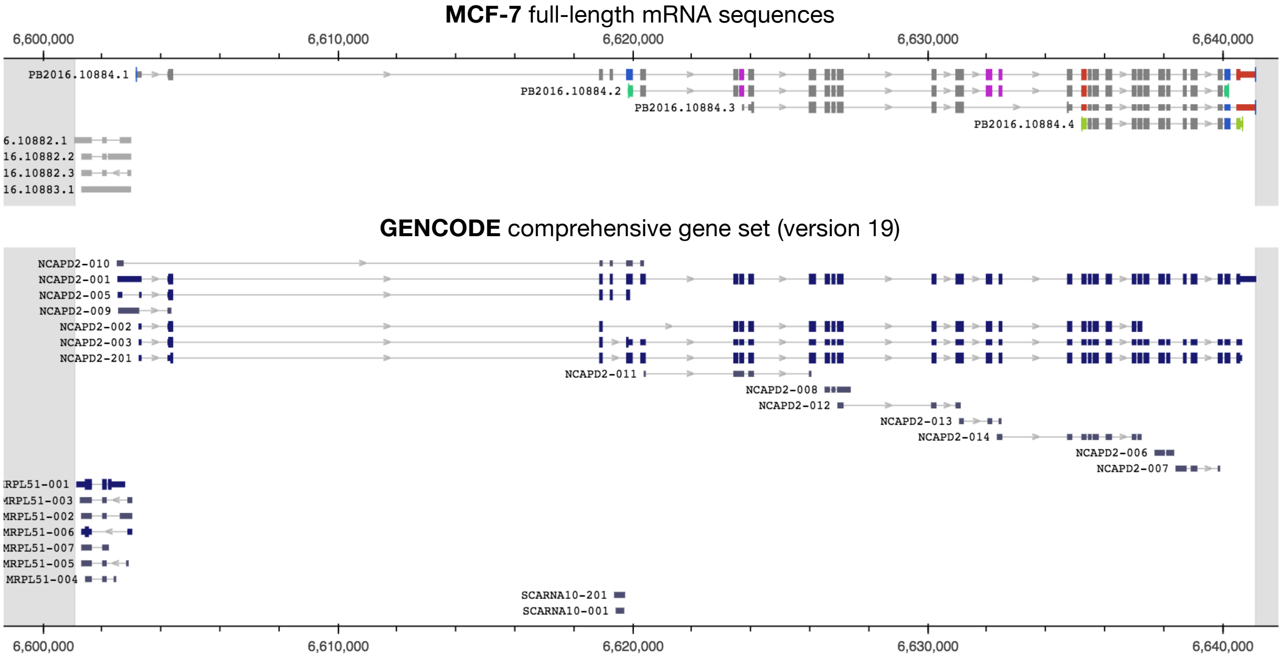

Figure S9 – The interdependence of transcription and mRNA processing of *NCAPD2*. Alternative transcription start sites are linked to the usage of alternative polyadenylation sites in three separate incidences. In addition, multiple interdependencies between alternative splicing of exons are depicted in different colors. For instance, alternative TSS and PAS observed in PB2016.10884.2 transcript are found to be mutually inclusive, illustrated in green. In addition, coupling events between series of exons are illustrated in purple as they are part of an interconnected subnetwork.

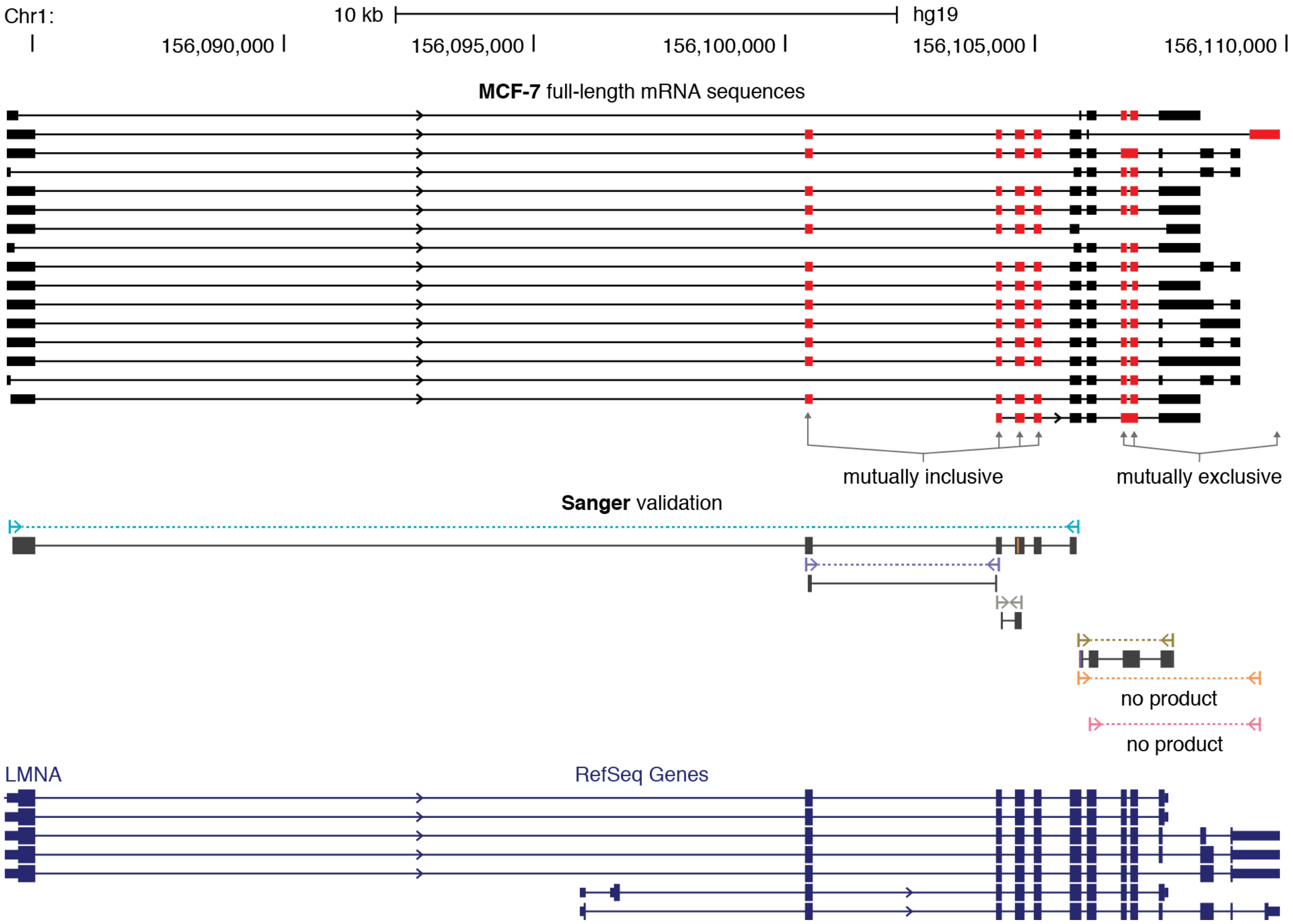

Figure S10 – The interdependence of transcription and mRNA processing of *LMNA*. Alternative consecutive exons are linked. In addition, A set of alternative exons are mutually exclusive to the usage of the most distal alternative polyadenylation site. Targeted Sanger sequencing supports the mutual inclusive and exclusive events depicted in red.

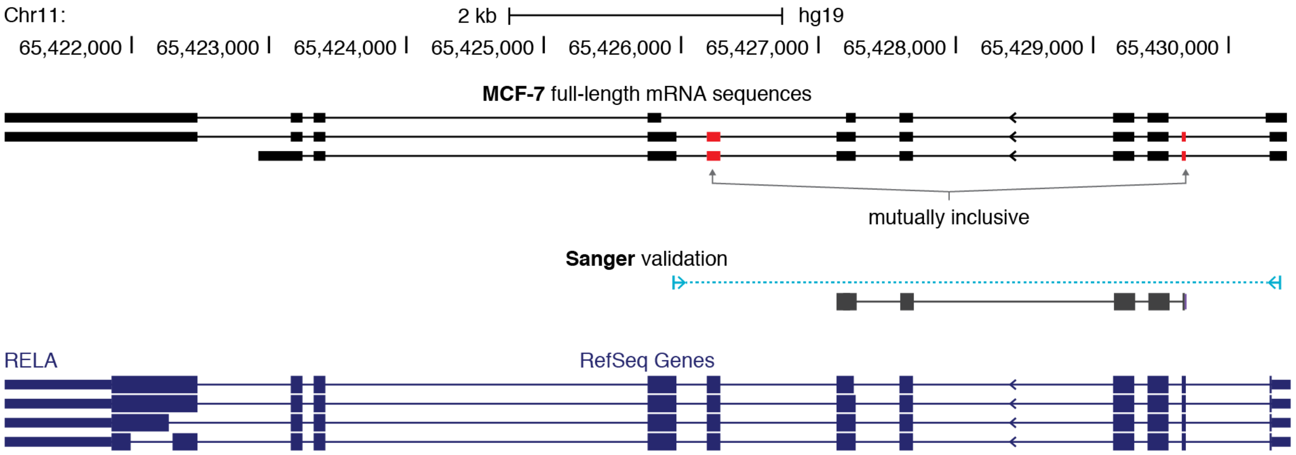

Figure S11 – The interdependence of transcription and mRNA processing of *RELA*. Mutual inclusivity of distant alternative exons, depicted in red. Targeted Sanger sequencing partially supports the mutual inclusive and exclusive events depicted in red. Due to the size of the amplified fragment, Sanger sequence has low quality at either end that results in loss of sequence and partial alignment to the genome.

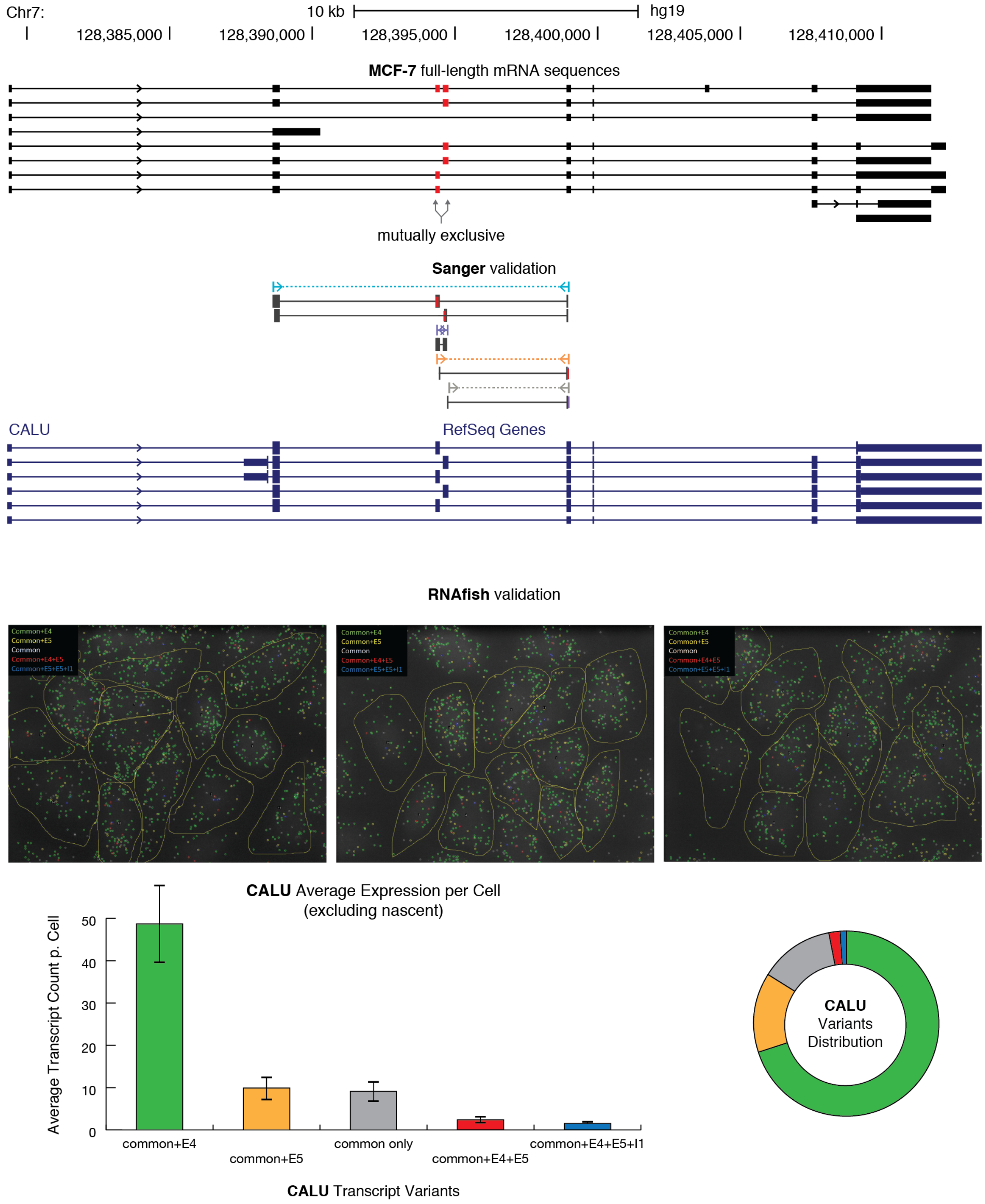

Figure S12 – The preferential interdependence of transcription and mRNA processing of *CALU*. Mutual exclusivity of consecutive alternative exons, depicted in red. Targeted Sanger sequencing supports the mutual exclusive splicing event. In addition, smRNA-fish experiment (common + exon 4: green circles; common + exon 5: yellow circles; common exons only: white circles; and common + exon 4 and 5: red circles; as well as all four in blue circles) shows preferential mutual exclusivity of alternative exons at single cell and single molecule level.

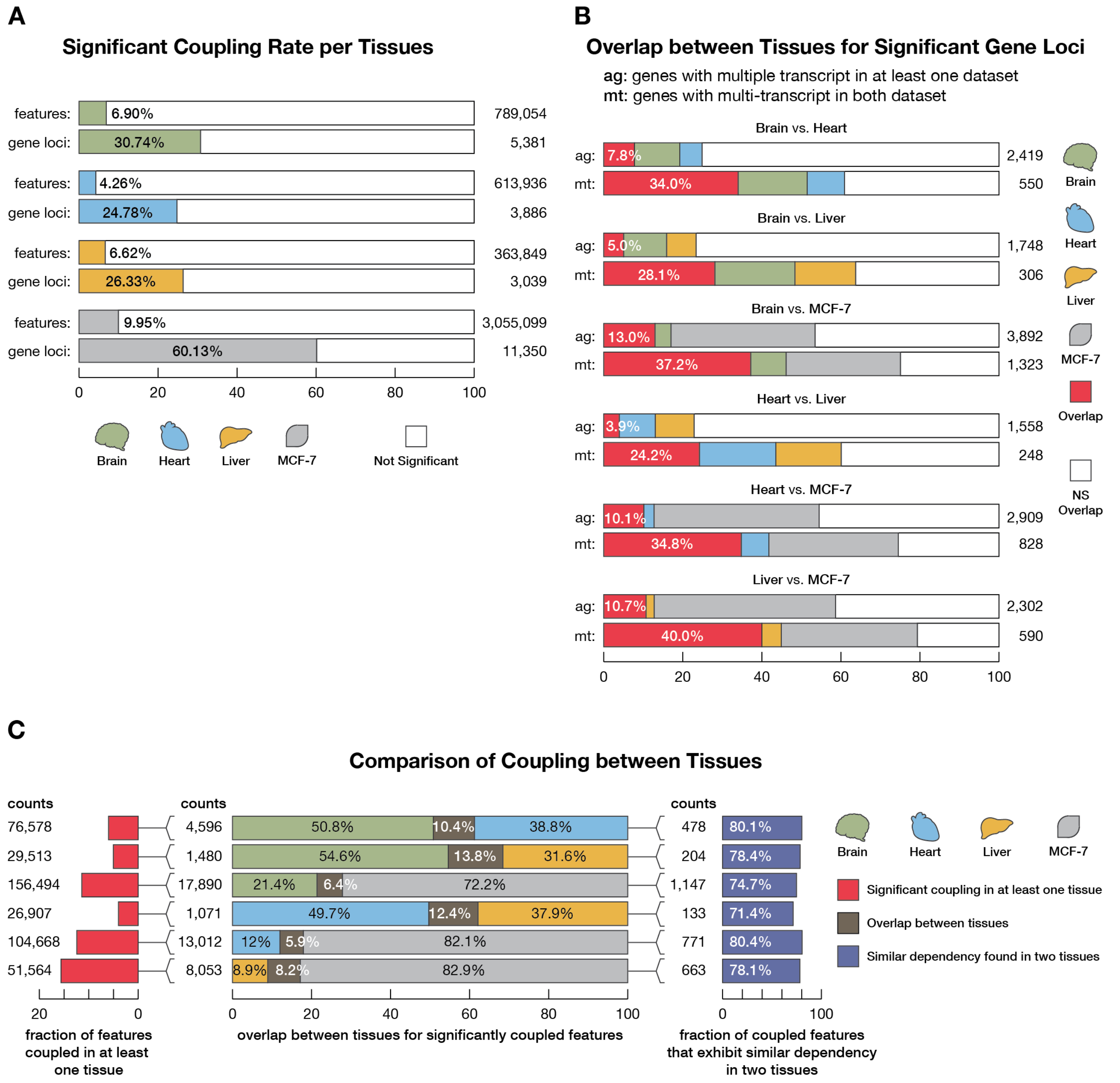

Figure S13 – Conservation of interdependencies across multiple human tissues. **A)** Bar plots show the proportion of feature-pairs or gene loci with multiple transcripts that exhibit coupling in full-length RNA sequencing data from three human tissues and MCF-7 cells. Each dataset is color coded. **B)** Bar charts illustrate the proportion of genes (ag) or genes with multiple transcripts (mt) that exhibit in multiple samples (red) as well as the proportion of genes that exhibit sample-specific coupling. **C)** Bar charts depict the number of feature-pairs that are significantly coupled in at least one dataset (red). The proportion of feature-pairs that are coupled in multiple tissues are shown in the middle bar chart along with the proportion of coupled feature-pairs in multiple tissues with the same type of dependency (blue bar chart).

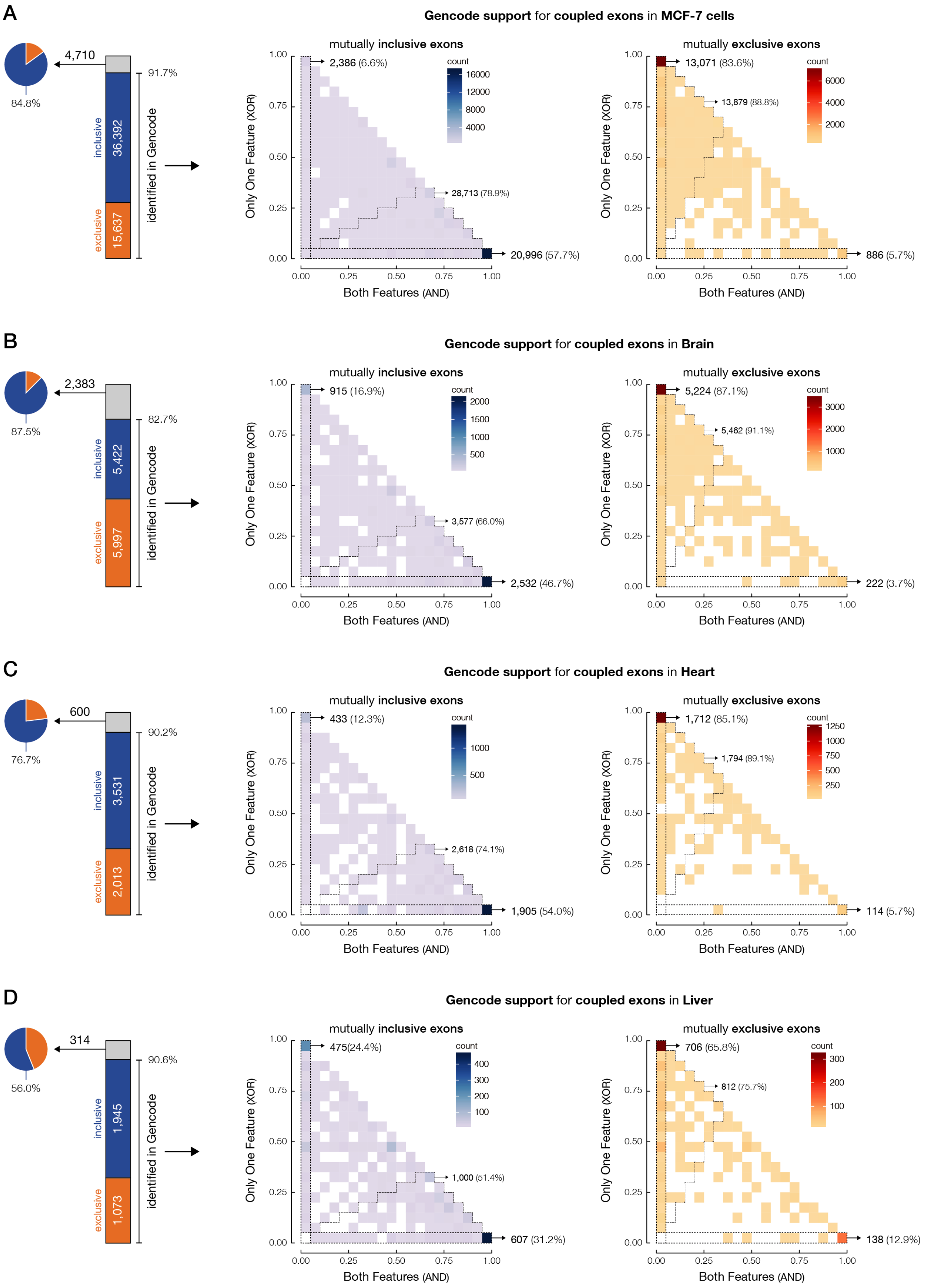
A
Gencode support for coupled exons in MCF-7 cells
4,710
84.8%
91.7%
inclusive
36,392
exclusive
15,637
identified in Gencode
mutually inclusive exons
2,386 (6.6%)
28,713 (78.9%)
20,996 (57.7%)
count
16000
12000
8000
4000
mutually exclusive exons
13,071 (83.6%)
13,879 (88.8%)
886 (5.7%)
count
6000
4000
2000
Only One Feature (XOR)
Both Features (AND)
0.00
0.25
0.50
0.75
1.00
B
Gencode support for coupled exons in Brain
2,383
87.5%
82.7%
inclusive
5,422
exclusive
5,997
identified in Gencode
mutually inclusive exons
915 (16.9%)
3,577 (66.0%)
2,532 (46.7%)
count
2000
1500
1000
500
mutually exclusive exons
5,224 (87.1%)
5,462 (91.1%)
222 (3.7%)
count
3000
2000
1000
C
Gencode support for coupled exons in Heart
600
76.7%
90.2%
inclusive
3,531
exclusive
2,013
identified in Gencode
mutually inclusive exons
433 (12.3%)
2,618 (74.1%)
1,905 (54.0%)
count
1000
500
mutually exclusive exons
1,712 (85.1%)
1,794 (89.1%)
114 (5.7%)
count
1250
1000
750
500
250
D
Gencode support for coupled exons in Liver
314
56.0%
90.6%
inclusive
1,945
exclusive
1,073
identified in Gencode
mutually inclusive exons
475(24.4%)
1,000 (51.4%)
607 (31.2%)
count
400
300
200
100
mutually exclusive exons
706 (65.8%)
812 (75.7%)
138 (12.9%)
count
300
200
100

Figure S14 – Gencode support for preferentially interdependent exons in the MCF-7 cells and three primary human tissues. Bar chart shows the proportion of coupled exons (mutually inclusive in blue and mutually exclusive in orange) that were found in Gencode annotation. Heat maps show density of the fraction of transcripts that contain both coupled exons (AND) versus the fraction of transcripts that only contain one of the two alternative exons (XOR). Mutually inclusive (Blue) and mutually exclusive (Orange) exons were plotted separately as opposite trend is expected under the assumption that many of the interdependencies found in MCF-7 cells **(A)** and human brain **(B)**, heart **(C)**, and liver **(D)** tissues are already known. Vertical dotted line depicts the number of coupled exons that could only be found exclusively in the Gencode, without any evidence of them being annotated in the same Gencode transcript. Horizontal dotted line depicts the number of coupled exons that were predominantly annotated in the same transcripts and not found exclusively in the Gencode. The area with over two-fold greater number of transcripts supporting the inclusion (left heat map) or exclusion (right heat map) of coupled exons are also illustrated.

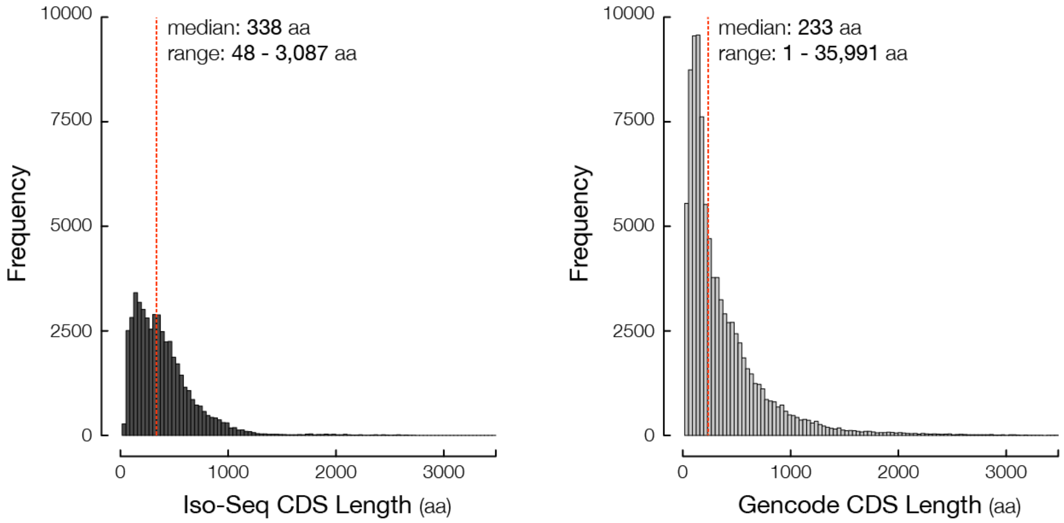

Figure S15 – Distribution of the length of predicted open-reading frames in MCF-7 cells. Histograms show the length of predicted coding sequences in MCF-7 cells (left panel) along with those annotated in Gencode (right panel). The x-axis is in amino acids (aa) scale. We have not applied any filtering on Gencode annotation to remove very short ORFs. The number of ORFs that are shorter than 10aa and 50aa are 285 and 5407, respectively.

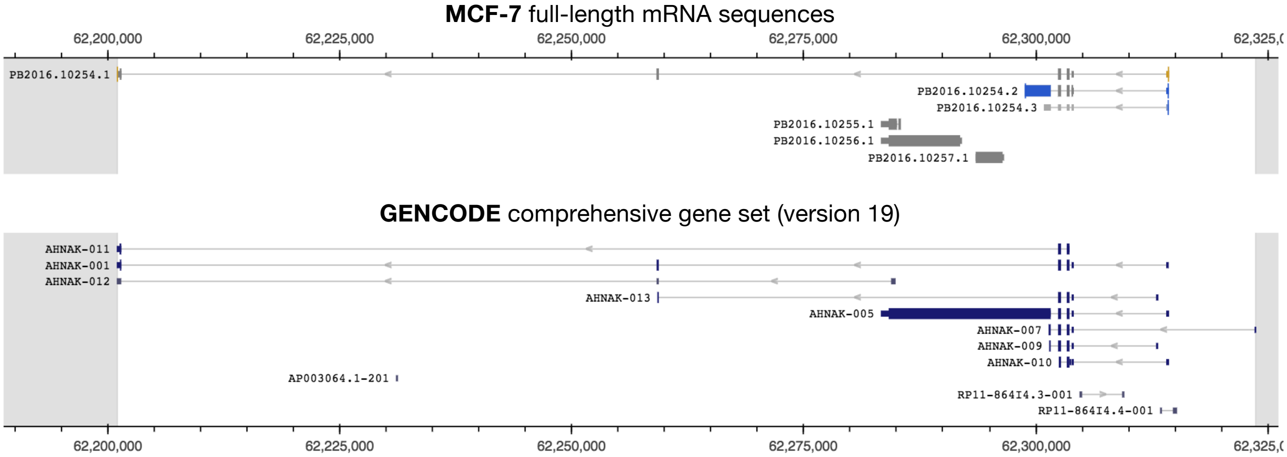

Figure S16 – Identified *AHNAK* transcripts in MCF-7 cells using full-length mRNA sequencing. The central domain of AHNAK protein containing 128aa repeat (located within the longest exon annotated in Gencode) is represented by two single-exon transcripts and is mostly absent in multi-exon transcripts.

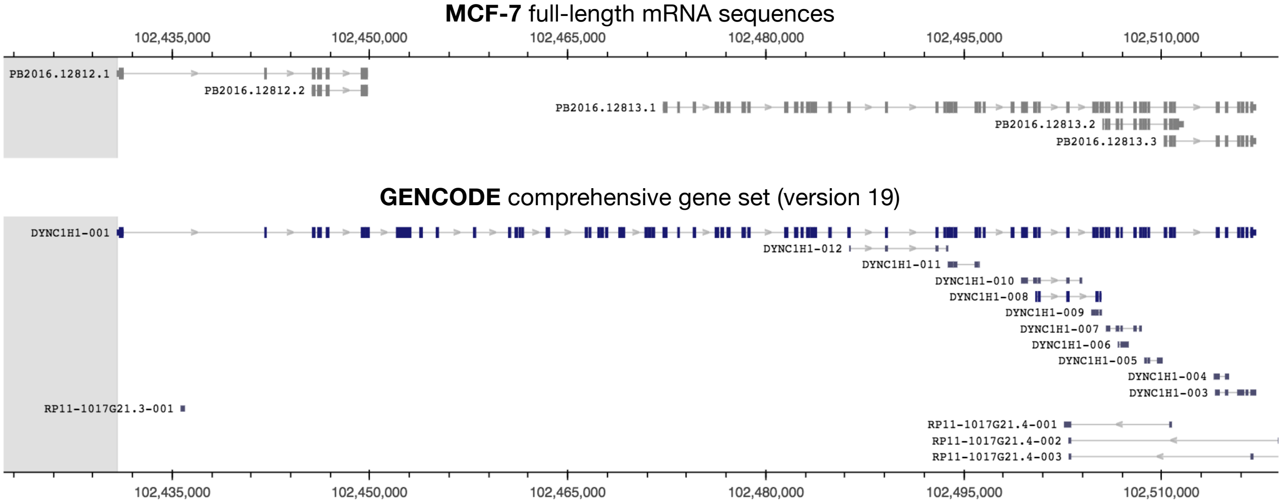

Figure S17 – Identified *DYNC1H1* transcripts in MCF-7 cells using full-length mRNA sequencing. This gene is represented by two sets of transcripts covering the 5' and 3' end of the gene, annotated in Gencode. It is likely that the canonical transcript is not captured by PacBio sequencing due to the size of the RNA molecule and limitations of full-length cDNA synthesis and sequencing.

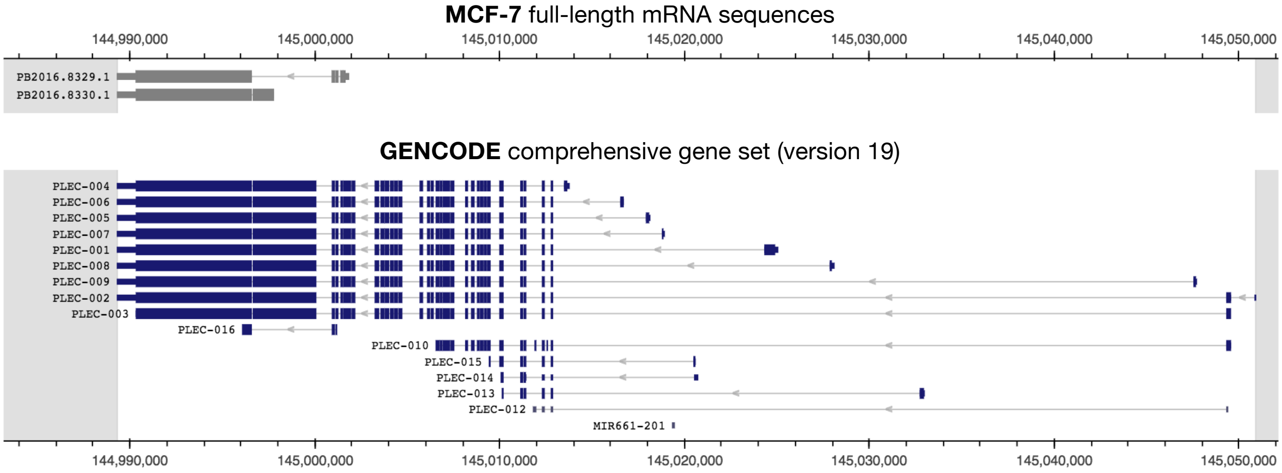

Figure S18 – Identified *PLEC* transcripts in MCF-7 cells using full-length mRNA sequencing. Transcripts of this gene are not well captured in PacBio data due to the size of this gene and limited range of full-length cDNA synthesis and sequencing.

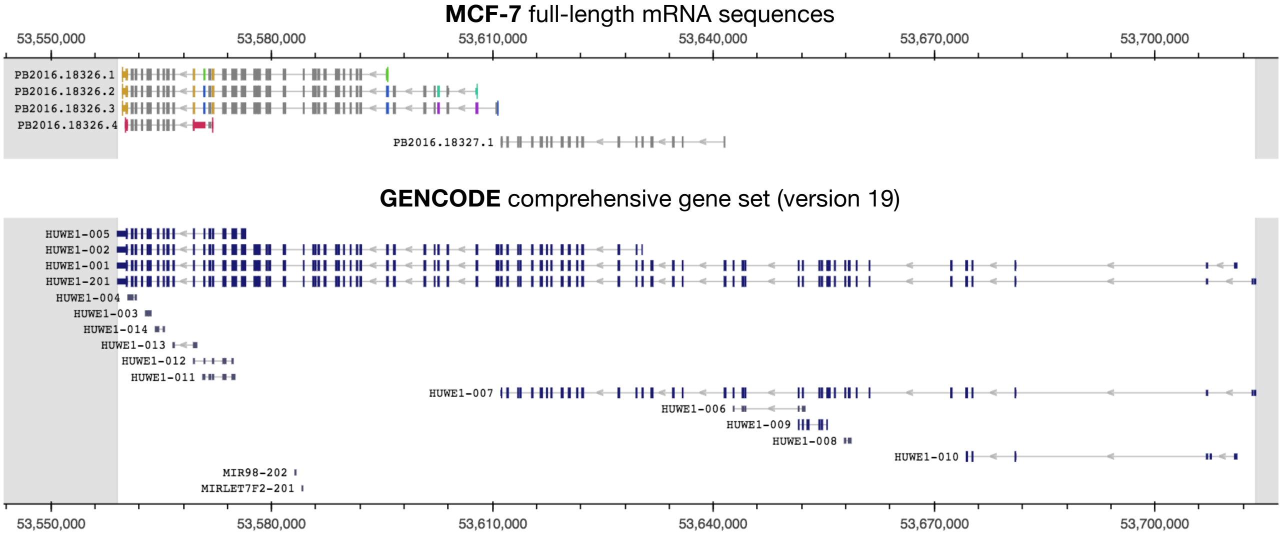

Figure S19 – Identified *HUWE1* transcripts in MCF-7 cells using full-length mRNA sequencing. PacBio transcripts do not represent the 5'-end of the gene, which may be due to suboptimal first strand cDNA synthesis to reach the true 5'-end.

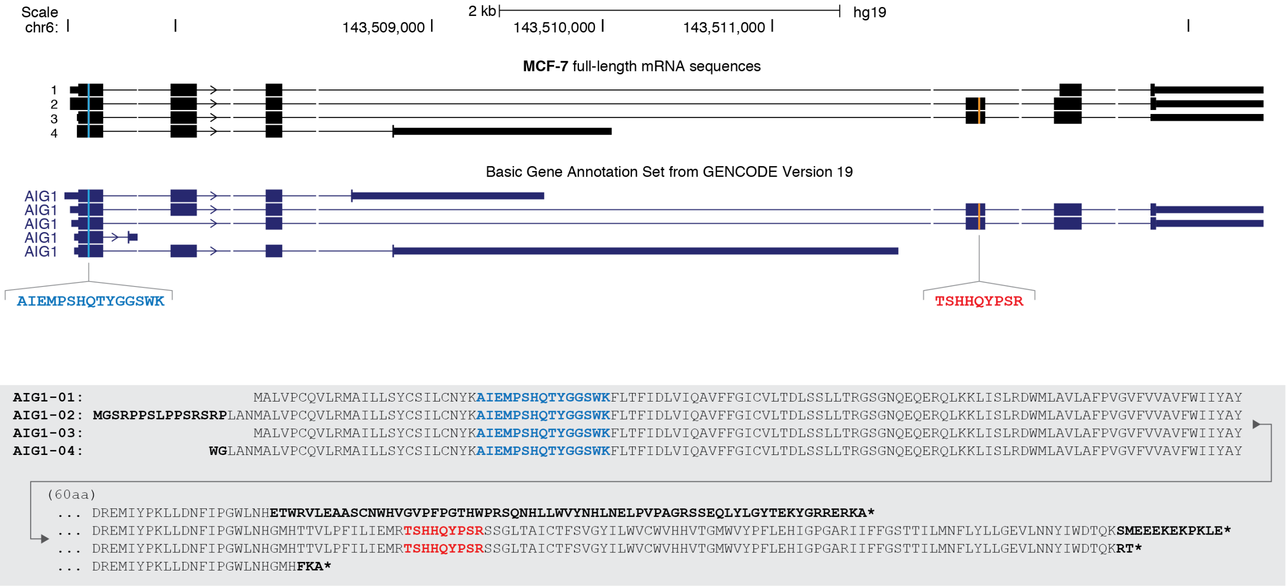

Figure S20 – Classification of peptide matches based on their specificity. Single-gene peptide shown in blue represents peptide hits that are associated with all coding transcripts of a given gene, whereas sub-gene peptide shown in red represents peptides that only hit a subset of coding transcripts and therefore provide a higher specificity.

Table S3 – The comparison of detected peptides based on PacBio and GENCODE v.19 annotation. Different classes of peptide matches: single-transcript hits representing peptides that hits genes with only one annotated transcript; sub-transcripts hits defining peptides that hit a subset of transcripts from a given gene; all-transcripts hits represent peptides that match all transcripts of a given gene; multi-gene hits are those peptides that match to more than one gene. Percentages are calculated in relation to the total number of peptides represented per each category based on Gencode annotation.

		Gencode v.19				
		**Single transcript** (2,637)	**Sub-transcripts** (28,857)	**All-transcripts** (4,820)	**Multi-gene** (1,956)	**No hit** (358)
**PacBio**	**Single transcript** (3,588)	**18.0%** (475)	8.0% (2,307)	13.8% (668)	4.3% (85)	14.8% (53)
	**Sub-transcripts** (23,113)	45.9% (1,209)	**64.8%** (18,711)	50.1% (2,415)	26.4% (516)	73.2% (262)
	**All-transcripts** (6,739)	17.6% (465)	17.1% (4,930)	**23.1%** (1,114)	10.5% (205)	7.0% (25)
	**Multi-gene** (2,316)	2.8% (73)	3.4% (981)	4.1% (196)	**53.6%** (1,048)	5.0% (18)
	**No hit** (2,872)	15.7% (415)	6.7% (1,928)	8.9% (427)	5.2% (102)	**0.0%** (0)

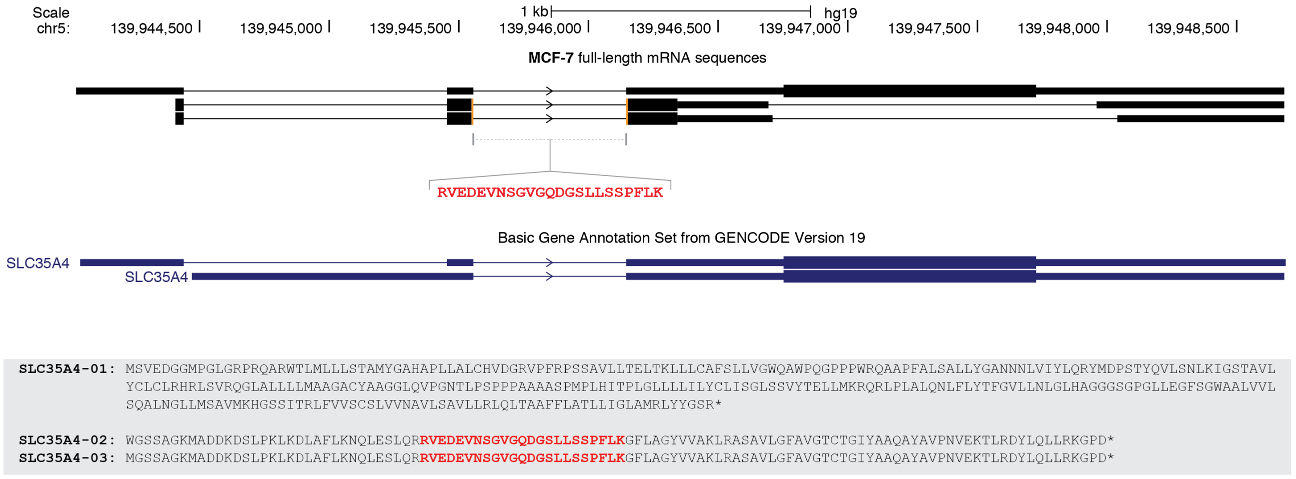

Figure S21 – Novel peptide match in SLC35A4. Peptide spanning exon-exon junction is depicted in red.

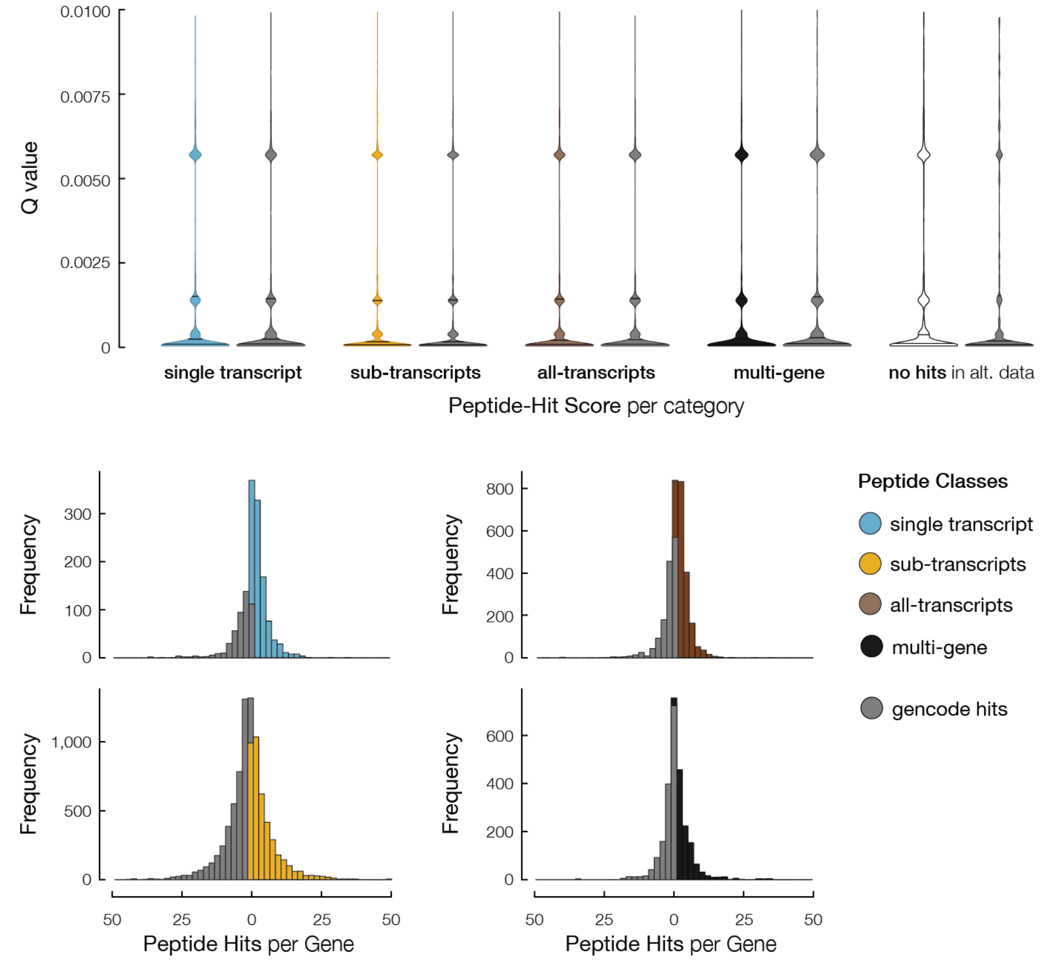

Figure S22 – Significance of peptide matches based on annotation. Distribution of q-values for peptide matches based on each category and annotation used is depicted along with histograms of the number of hits per gene. Colors depicts different peptide hit categories.

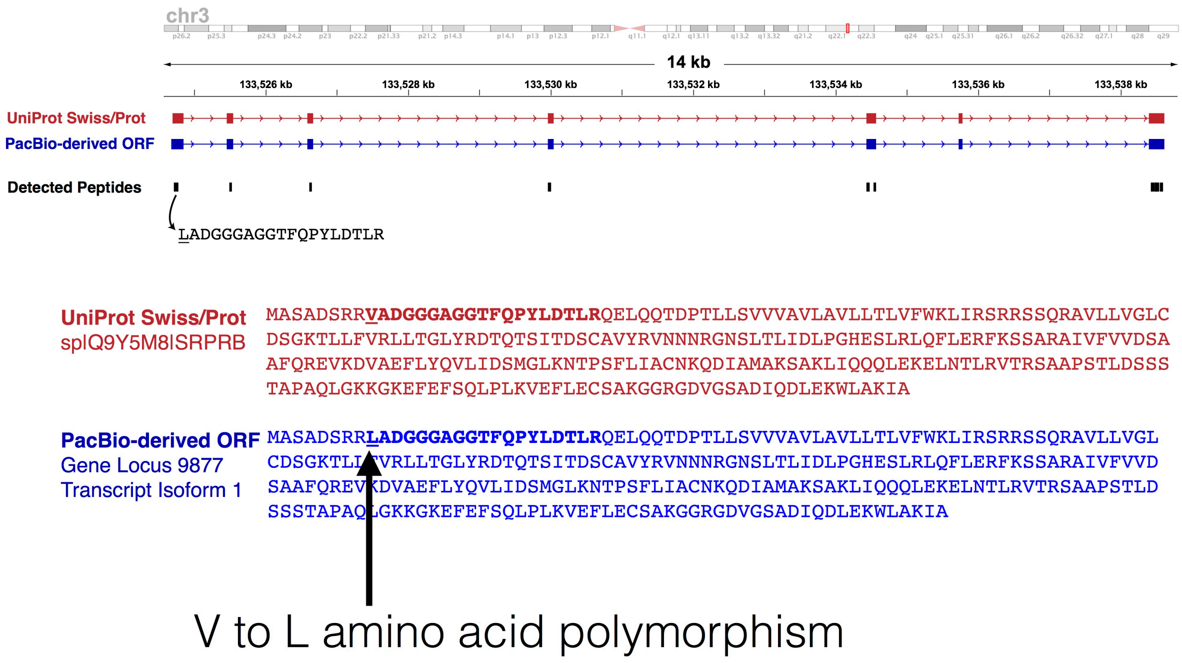

Figure S23 – Single amino acid substitution (SAS) events in MCF-7 cells result in loss of peptide assignment. Peptide sequence shown in bold represent amino acid sequence that could not found in Gencode due to the presence of SAS. However, PacBio derived ORF can capture sample-specific differences and therefore provides a match for the probed peptide.

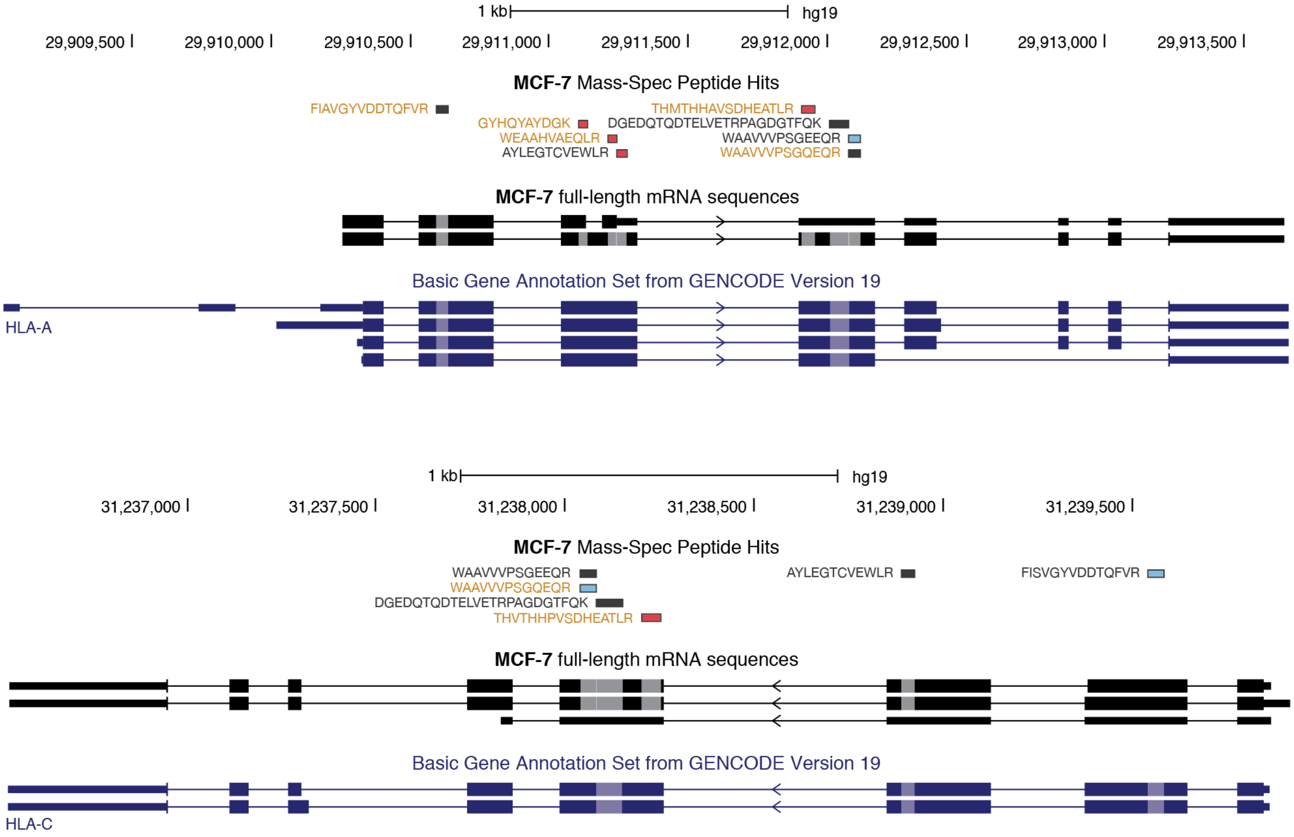

Figure S24 – Misalignment of peptides in HLA-A and HLA-C loci due to single amino acid substitutions. Peptides shown in red represent amino acid sequences that were not found in Gencode. Black peptides are multi-gene peptides that are found in both sets. Light blue peptides represent misaligned peptides due to the presence of single amino acid substitution (SAS).

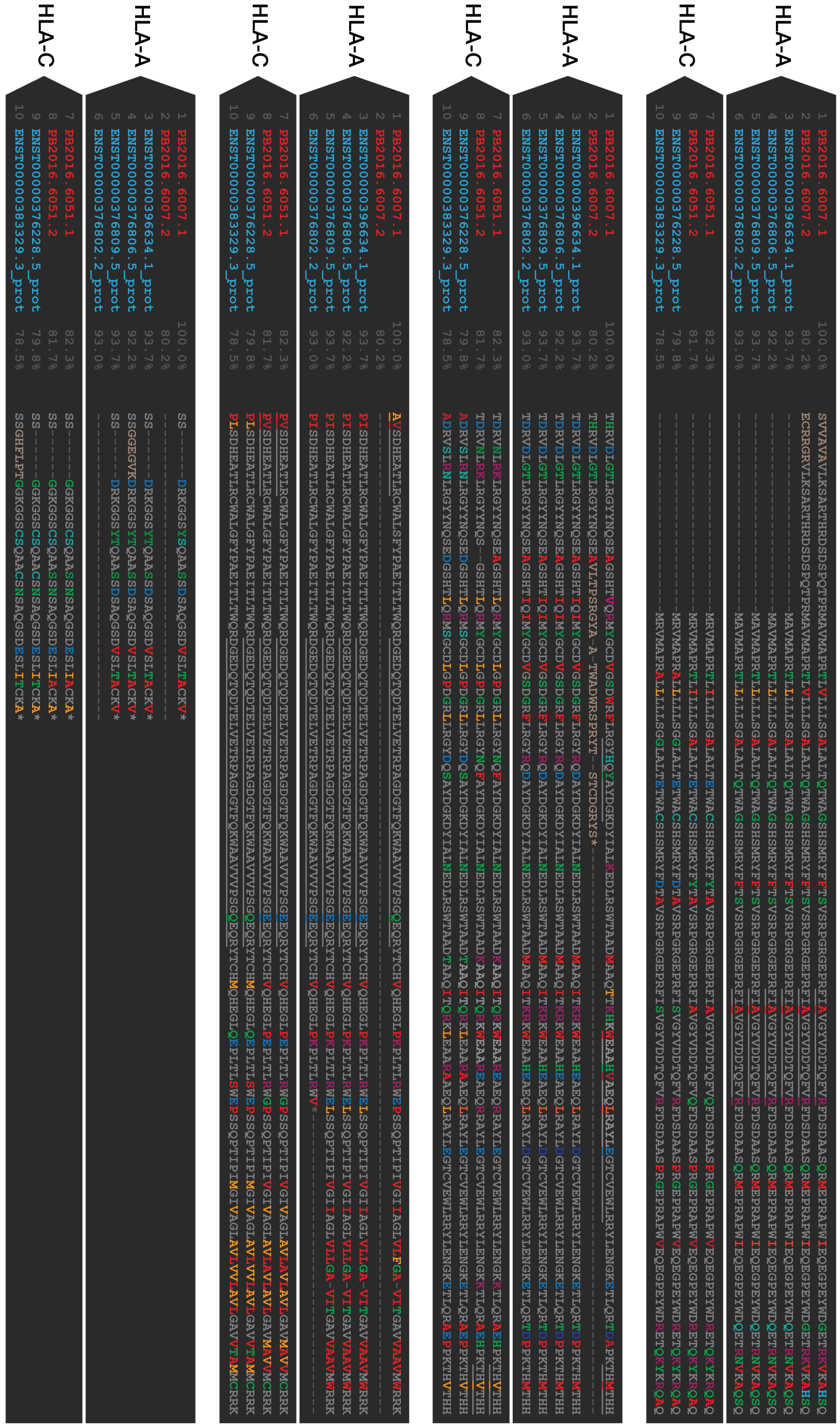

Figure S25 – Multiple alignment of ORFs for HLA-A and HLA-C genes along with peptide matches. Colored amino acids represent sequence differences between ORFs, some of which may affect alignment of peptides. Both PacBio and Gencode ORFs are shown.

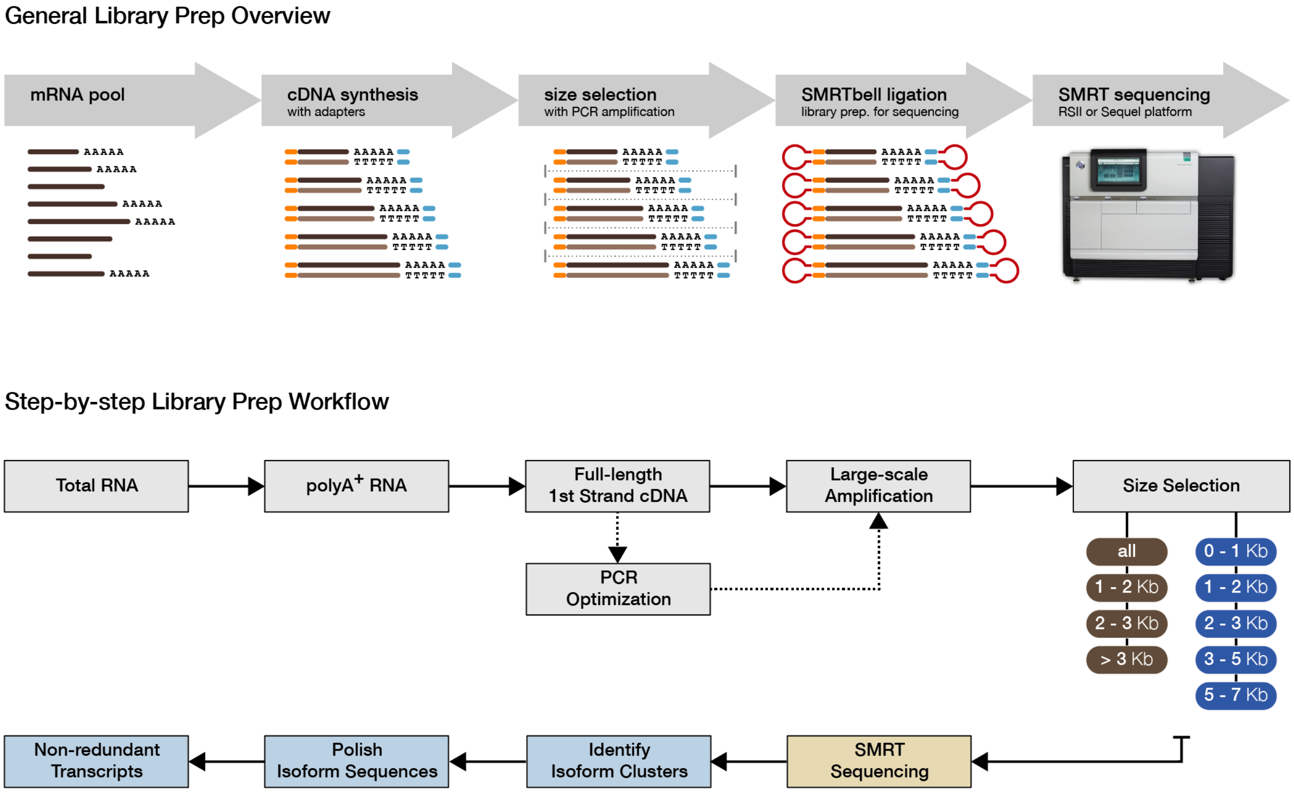

Figure S26 – The schematic overview of the general and step-by-step workflow for the library preparation, sequencing and data processing.
