# Supplementary material for: Full-length mRNA sequencing uncovers a widespread coupling between transcription initiation and mRNA processing
Source: Genome Biol. 2018 Mar 29;19:46. doi: 10.1186/s13059-018-1418-0 (PMC5877393; doi:10.1186/s13059-018-1418-0)
Supplement: Supplementary file 2 — Reviewer reports and authors’ response to reviewers. (DOCX 30 kb) [file 13059_2018_1418_MOESM2_ESM.docx]

**First round of review**

Reviewer 1 report:

**Are the methods appropriate to the aims of the study, are they well described, and are necessary controls included?**

The methods were difficult to assess.

**Are the conclusions adequately supported by the data shown**?

It is not clear from the presented methods whether the conclusions are justified.

**Are sufficient details provided to allow replication and comparison with related analyses that may have been performed?**

The analysis details were too superficial to be replicated.

**Does the work represent a significant advance over previously published studies?**

The work could potentially represent a major advance.

**Is the paper of broad interest to others in the field, or of outstanding interest to a broad audience of biologists?**

Yes: The demonstration that splicing events are coupled could be of high interest.

**Comments**

In their paper entitled "Full-length mRNA sequencing uncovers a widespread coupling between transcription and mRNA processing", Anvar and colleagues examine coupling between splicing and mRNA processing events in deeply sequenced full length Pacific Bioscience data. In so doing, the authors generate a broad range of data analyses and some validations to assess correlations between alternative transcription, splicing and polyadenylation in MCF-7 cells. In addition, the authors examine these processes in three primary tissue types. Finally, the authors use shotgun proteomics to validate novel ORFs.

Overall, the technology utilized in this manuscript and the questions addressed are potentially powerful and of high interest. In particular, the demonstration that specific combinations of transcription, splicing and polyadenylation events are overrepresented in the data is quite intriguing. However, the methods used to draw these conclusions are often unclear, and the validations are not sufficient to support all the claims. In the end, the message becomes muddled with both the biology and analysis insufficiently addressed.

Specific comments

1)	Manuscript throughout. It would benefit the authors if they could pull CAGE/RNA-seq/PAS-seq data to form at least local event validation for this dataset. Likewise, some biochemical validation of the observed events (beyond the FISH) is required (example, splicing gels and PAS mapping).

2)	Results p1, line 22. The minimum correlation coefficient among 5 public datasets is 0.69 in Fig S2A, not 0.74 as stated in results. I am not convinced that these correlations represent reliable locus-level quantification.

3)	Fig 1 (Results p1). In the description of the coupling calculation, it is not clear that the Fisher's exact test as constructed is the appropriate test, or whether it will detect interdependency. For example, the authors fail to mention if constitutive exons would also be defined as coupled.

4)	The authors find 7,364 single exon transcripts and mention transcripts of 54bp in length; authors should indicate whether this value is equivalent to other RNA-seq methods.

5)	line 59: The authors describe the observed interdependencies to be a novel advantage arising from their sequencing result, but it is unclear what fraction of them are already known. For example, rather than using a merge of all gencode features, are known interdepencies already encoded in the annotated transcript models?

6)	Fig 2 C-E, Results p.2. A p-value cutoff is more appropriate to include rather than just the label 'significant.'

7)	Fig 3, Results p. 2. There is no demonstration that PASs are accurately mapped. The methods state that they are curated to remove noise, but this is vague. For example, the authors find that almost half of all alternative polyadenylation sites are found in their dataset do not have a canonical polyA site. The authors should demonstrate using alternative methods for polyA site usage that this is a common occurrence as validation.

8)	Results p. 2, line 52: The authors state that their PAS result 'partly disagrees' with previous observations of UTR shortening, but this comparison is unclear and under-developed. This also highlights the utility of comparative analysis, to provide a baseline to identify differences in UTR length or coupling.

9)	Results, p. 3, line 4. Proportion differences are stated as 'significant,' but no statistical analysis is performed.

10)	Results p.3, line 20. In their RBP analysis, the authors identify RBP motifs near alternative exons, but this is unsurprising and perfunctory.

11)	Fig 4, Results p.4. Proteomics analysis is a good approach to demonstrate the value of their methodology, but it is not clear that the comparison being made demonstrates this. The comparison is made to annotated Gencode transcripts, which is not a sample-specific set of transcript models. This point is also made in the last paragraph of the discussion. Whether the unannotated peptides discovered by the PacBio data would not also be identified by standard short-read sequencing is unclear. This analysis also seems to revolve around novelty of isoforms versus coupling making this part of the manuscript slightly confusing to follow.

12)	Methods. A significant amount of the methods are given as URLs. The methods for data generation are located on github repository rather than fully explained or cited.

In sum, the technology used is potentially very useful, and the novel analysis described is creative. However, the manuscript is lacking in adequate evidence that their sequencing depth is sufficient, that quantification is accurate, and that the computational method is valid. The results are qualitative in nature, and there is a lack of rigor on quantitative statements and statistics. A sufficient demonstration of an advantage over currently available methods is lacking. The coupling calculations would be more useful if they could be demonstrated to find quantitative differences between samples. Finally, the proteomics data feel tangential to the "coupling" aspect of the manuscript.

Reviewer 2 report:

**Are the methods appropriate to the aims of the study, are they well described, and are necessary controls included?**

yes

**Are the conclusions adequately supported by the data shown**?

Don’t know

**Are sufficient details provided to allow replication and comparison with related analyses that may have been performed?**

Not sure

**Does the work represent a significant advance over previously published studies?**

Not sure

**Is the paper of broad interest to others in the field, or of outstanding interest to a broad audience of biologists?**

Yes: This very interesting paper, using long read sequencing data to investigate the connectivity of exons and relationships to alternative promoters and terminators, is very timely and should be transformative for the gene expression community. The main contribution is in the analysis of a massive amount of long read RNA-Seq data obtained by scientists working at Pacific BioSciences.

**Comments**

This very interesting paper, using long read sequencing data to investigate the connectivity of exons and relationships to alternative promoters and terminators, is very timely and should be transformative for the gene expression community. The main contribution is in the analysis of a massive amount of long read RNA-Seq data obtained by scientists working at Pacific BioSciences. In its present form, this paper is unpublishable because the computational approaches are not well explained. It is impossible for a molecular biologist to read and appreciate. The main task before the authors is to achieve clarity about what the authors did bioinformatically. Additional suggestions are listed below:

Issues about the writing. Some terms are non-standard and make the text unclear. For example, "alternative transcription" is used a lot. This is not a thing. Presumably the authors are referring to alternative transcription start sites or alternative promoters. When the authors use the term "single transcript", we are not sure if this means there is only 1 transcript for that gene or if there is only 1 discrete transcript isoform (of which there may be many). Several paragraphs, e.g. ones referring to data presented in the Supplement, are impossible to read without looking at the figures. In other words, the text cannot be understood on its own. Instead, readers have to work backwards from looking at the data.

Experimental details are missing. In this work 147 SMRT cells of data were generated. Presumably, these were prepared on different days in batches. How do the authors handle batch biases (e.g. see Leek et al PMID 20838408? This is important for anyone intending to use this platform for a large number of reads. Although some data is shown in the supplement, please state clearly in the main text or in a main figure how many mappable reads were obtained, and how many transcripts per gene on average. Finally, we did not understand how "polished reads" are obtained. They appear to be partial reads that are filled in by the analysis tool. How can this be valid data?

The validation of the CALU transcript isoforms done by FISH and shown in the supplement is consistent with mutually exclusive exons. However, the experiment does not validate use of an alternative promoter. This is an important aspect of the work.

There is a lot of discussion and motif finding that suggests the activity of an APA motif, which may be bound by MBNL. Some kind of experimental validation is required for this, due to the redundancy of motifs bound by the hundreds of RNA binding proteins expressed by cells.

**Authors’ response to reviews**

Referee 1:

1.Overall, the technology utilized in this manuscript and the questions addressed are

potentially powerful and of high interest. The demonstration that specific combinations

of transcription, splicing and polyadenylation events are overrepresented in the data is

quite intriguing. However, the methods used to draw these conclusions are often

unclear, and the validations are not sufficient to support all the claims.

Response: The description of methods that are used in this study is provided in the

main text and supplementary material and illustrated in Figure 1. However, we

appreciate that readers may benefit from additional information regarding the

developed strategy to further improve the clarity of the approach and interpretation of

the findings.

We have now revised the manuscript and provided additional information on the

analyses performed in this study. We have also provided additional supporting material

in the supplementary document. Please note that all the source codes used in this

study is readily provided along with detailed documentation in a GitHub and GitLab

repositories.

Additional supporting evidence for the claims is provided below and in the revised

manuscript.

2.Manuscript throughout. It would benefit the authors if they could pull CAGE/RNAseq/

PAS-seq data to form at least local event validation for this dataset. Likewise,

some biochemical validation of the observed events (beyond the FISH) is required

(example, splicing gels and PAS mapping).

Response: It is not feasible to perform a thorough comparison between standard RNASeq

experiments and full-length mRNA sequencing due to inherent limitations of shortread

sequencing technologies. However, we agree with the reviewer that they may be

used for validation of local events such as the use of transcription start sites (TSSs)

and polyadenylation sites (PASs). We have performed a comparison of CAGE and

RNA-PET data sets from the Encode project for the MCF-7 cell line. The results of this

comparison have now been added to the manuscript (page 4, paragraph 4; Figure S3).

In short, the comparison of Encode CAGE data set with full-length mRNA sequencing

data revealed that the majority of identified TSSs in CAGE were in close proximity of

their counterpart in PacBio data set, with median distance of 1bp. In addition, 99.5% of

genes with multiple TSSs in CAGE have multiple TSSs identified in PacBio data.

However, we could identify 1,386 additional multi-TSS genes in PacBio.

RNA-PET captures the connections between 5’ and 3’ ends. This data was used for

the validation of the combinations of TSSs and PASs in the transcript structures

identified in PacBio data. Notably, the limited resolution of RNA-PET data needs to be

taken into account as the Encode RNA-PET dataset was curated to merge 5’ and 3’

tags that were located within 100bp of each other. The results of this comparison

further confirmed the validity of detected 5’ and 3’ ends of transcripts based on fulllength

mRNA sequencing as the majority of RNA-PET terminal sites were in close

proximity of their counterparts in PacBio data set, with median distances of 70bp and -

11bp (TSSs and PASs, respectively).

3.Results p1, line 22. The minimum correlation coefficient among 5 public datasets is

0.69 in Fig S2A, not 0.74 as stated in results. I am not convinced that these

correlations represent reliable locus-level quantification.

Response: Due to inherent limitations of short-read technologies, the only viable

comparison between datasets is to assess quantifications at gene level. To this end,

we have calculated the median coverage across the gene and correlated that with

what was detected using full-length mRNA sequencing. This comparison shows that

the locus-level quantifications are in good concordance with those produced using

standard RNA-Seq experiments.

As correctly stated by the reviewer, the minimum correlation coefficient among 5 public

datasets is 0.69. This is now corrected in the manuscript.

4.Fig 1 (Results p1). In the description of the coupling calculation, it is not clear that the

Fisher’s exact test as constructed in the appropriate test, or whether it will detect

interdependency. For example, the authors fail to mention if constitutive exons would

also be defined as coupled.

Response: Our strategy is based on examining alternative exons and alternative TSSs

and PASs. Thus, features that are present in all transcripts of a given gene were not

assessed. Therefore, constitutive exons were not evaluated for coupling. There are

multiple rules that were applied to ensure that identified interdependencies are

meaningful. We now provide additional explanation of how the test is performed below

and in the main text.

The two-by-two contingency table for pair of features is constructed based on the

following criteria: 1 – Two features should not partially or fully overlap; 2 – For TSSs,

features that are located in their upstream region are not compared as their exclusivity

is given. The same rule is applied to the downstream of PASs; 3 – Only transcripts that

fully cover the region that is represented by two features are used to populate the 2-by-

2 contingency table. This is to ensure that all counts are based on clear observation of

their inclusion or exclusion. 4 – Single-exon transcripts are excluded from the analysis

and only multi-transcript loci are examined for interdependencies. Finally, we have

applied Bonferroni multiple-testing correction to minimize the number of false positives

present in the final set. We believe that this strategy is designed to only capture

biologically meaningful interdependencies and we use the odds-ratio to differentiate

between mutual inclusivity and exclusivity.

5.The authors find 7,364 single exon transcripts and mention transcripts of 54bp in

length; authors should indicate whether this value is equivalent to other RNA-seq

methods.

Response: It is not possible to evaluate transcript lengths based on standard RNA-seq

experiments. This would require reliable reconstruction of transcript structures that is

limited by short length of sequencing reads. Instead, we have provided the name of the

matching Gencode transcripts for the shortest and longest transcript identified. The

shortest PacBio transcript (54bp) is already annotated in the Gencode v19 (ANKRD36-

004) while the longest transcript (10,792bp) represents a novel splice isoform for the

TAX1BP1 gene. We believe the strong concordance of Encode CAGE and RNA-PET

with MCF-7 full-length transcriptome provides further evidence for the validity of the

data used in this study.

From 7,364 single-exon transcripts identified in MCF-7 cells, 79% were found in gene

regions. However, only 9% of them have a match in Gencode. A reason for this lower

concordance in annotation is that such transcripts are hard to identify in standard RNAseq

and more classical experiments and therefore many such transcripts are still

missing from annotations. This is also reported in other independent studies such as

that of Kuo et al. (2017) – PMID: 28438136.

6.Line 59: The authors describe the observed interdependencies to be a novel

advantage arising from their sequencing results, but it is unclear what fraction of them

are already known. For example, rather than using a merge of all Gencode features,

are known interdependencies already encoded in the annotated transcript models?

Response: Given the fact that Gencode annotation does not provide transcript-level

quantifications, we are not able to perform comparable statistics to report the level of

overlap between the two sets. However, we agree with the reviewer that a comparison

between identified coupling events and the current Gencode annotation is valuable.

For each coupled exon-exon pair in MCF-7 cells and three primary human tissues, we

have identified the number of transcripts that support: 1) both exons in the same

transcript (AND); 2) only one of the two exons (XOR); and 3) the absence of both

exons (NOR). Terminal exons were excluded for this analysis. Obtained counts were

then normalized to the total number of transcripts per gene to facilitate comparison

between genes with different number of transcripts. It is important to note that we only

consider transcripts that encompass a region represented by two alternative exons.

The results of this comparison have now been added to the manuscript (page 7,

paragraph 3; Figure S13). For MCF-7 cells, over 8% of coupled exons, primarily

representing mutual inclusive exons (85%), could not be found in Gencode annotation.

From all identified mutually exclusive exons, 83.6% were not present in the same

Gencode transcript (XOR), supporting their mutual exclusivity. Out of all mutually

inclusive exons, almost 58% were present in the same Gencode transcript (AND).

Although the latter number seem low, we found that in 79% of the cases the number of

times mutually inclusive exons are annotated in the same Gencode transcripts is at

least two-fold higher than them being annotated independently. Moreover, only about

6% of coupled exons showed an opposite dependency based on Gencode annotation.

Overall, we observed similar patterns for coupled exons that were found in three

primary human tissues, but the confirmation rates were slightly lower, likely due to their

lower sequencing depth. The largest proportion of coupled exons that could not be

found in Gencode annotation was found in brain. Liver tissue was found to be the most

distinct sample in terms of Gencode support for coupled exons as larger proportion of

coupling events were found to have an opposite dependency in annotated transcripts.

7.Fig 2 C-E, Results p.2. A p-value cutoff is more appropriate to include rather than just

the label ‘significant’.

Response: We have modified the figure accordingly.

8.Fig 3, Results p.2. There is no demonstration that PASs are accurately mapped. The

methods state that they are curated to remove noise, but this is vague. For example,

the authors find that almost half of all alternative polyadenylation sites are found in their

dataset do not have a canonical polyA site. The authors should demonstrate using

alternative methods for polyA site usage that this is a common occurrence as

validation.

Response: Figure 3A shows that the majority of PASs (~72%) were indeed associated

with canonical or non-canonical polyA motifs. However, we argue that there is an

increase in the proportion of PASs with unknown PA motif (for which none of the

known motifs could be found) that are coupled with TSSs.

For the reliability of represented PASs in MCF-7 full-length transcriptome, please refer

to our response to point 2 of referee 1. The comparison between PacBio data and

Encode RNA-PET suggests that the identified PASs are within very close proximity of

those identified based on Encode RNA-PET experiment.

9.Results p.2, line 52: The authors state that their PAS result ‘partly disagrees’ with

previous observations of UTR shortening, but this comparison is unclear and underdeveloped.

This also highlights the utility of comparative analysis, to provide a baseline to identify differences in UTR length or coupling.

Response: The argument for shortening of 3’ UTRs was based on the reduced number

of tandem PASs that could be identified in MCF-7 as oppose to previously published

study. Since we have not fully investigated the actual shortening of the 3’ UTR in MCF-

7 cells, we have removed this section from the manuscript.

10.Results, p.3 line 4. Proportion differences are stated as ‘significant’, but no

statistical analysis is performed.

Response: We now only use the word ‘significant’ when the event was evaluated by a

statistical test.

11.Results p.3, line 20. In their RBP analysis, the authors identify RBP motifs near

alternative exons, but this is unsurprising and perfunctory.

Response: The aim of this analysis was not to suggest that there will be RBP motifs

near alternative exons. This analysis was performed to identify what motifs were

enriched near coupled exons and PASs. Therefore, alternative exons and PASs that

do not show any coupling were used as a background set. This analysis provides a list

of potential RBPs that could be studied in follow-up experiments to unravel how

interdependencies are regulated during mRNA processing.

12.Fig 4, Results p.4. Proteomics analysis is a good approach to demonstrate the

value of their methodology, but it is not clear that the comparison being made

demonstrates this. The comparison is made to annotated Gencode transcripts, which is

not a sample-specific set of transcript models. This point is also made in the last

paragraph of the discussion. Whether the unannotated peptides discovered by the

PacBio data would not also be identified by standard short-read sequencing is unclear.

This analysis also seems to revolve around novelty of isoforms versus coupling making

this part of the manuscript slightly confusing to follow.

Response: The analysis of peptides relies on available open-reading frames to identify

those that perfectly match peptide sequences. Standard RNA-Seq may aid

accommodating for sample-specific genetic variations but does not help decipher

alternative, full-length open-reading frames. In this section, we have predicted openreading

frames based on full-length mRNA sequences that was subsequently followed

up with matching of peptides. Discrepancies in results reflect the limitations of using

annotations such as Gencode to identify and characterize peptides. We also show that

many peptides are mapped to interdependent exons and, therefore, can be expected

to be derived from the same protein isoform. This section is now revised to improve the

clarity and highlight the impact of these observations.

13.Methods. A significant amount of the methods are given as URLs. The methods for

data generation are located on github repository rather than fully explained or cited.

Response: All methods that were used in this study are provided as open-source and

shared via standard frameworks such as GitHub and GitLab repositories. Sharing

source codes through such repositories is considered as good research practice by the

community to ensure long-term maintenance, reproducibility, accessibility, and

interoperability. All the methods are now also described in detail in the supplementary

and the main text.

Referee 2:

1.The main contribution is in the analysis of a massive amount of long read RNA-Seq

data obtained by scientists working at Pacific Biosciences. In its present form, this

paper is unpublishable because the computational approaches are not well explained.

Response: We now provide additional information regarding the computational

approaches used and have also included supporting literature to improve the clarity.

The computational method has been described in the Methods section. The method

has been described in detail in a previous publication (Gordon et al., 2015) and has

been made available both in a software package (PacBio’s SMRTAnalysis software)

and as a stand-alone source code that is publicly available at GitHub repository.

2.Issues about the writing. Some terms are non-standard and make the text unclear. …

Several paragraphs, e.g. ones referring to data presented in the Supplement, are

impossible to read without looking at the figures. In other words, the text cannot be

understood on its own.

Response: The manuscript is now revised to avoid the use of non-standard

terminologies when possible. We have now revised the manuscript to improve the

readability.

3.Experimental details are missing. In this work 147 SMRT cells of data were

generated. Presumably, these were prepared on different days in batches. How do the

authors handle batch biases (e.g. see Leek et al PMID 20838408)? … Although some

data is shown in the supplement, please state clearly in the main text or in a main

figure how many mappable reads were obtained, and how many transcripts per gene

on average. Finally, we did not understand how “polished reads” are obtained. They

appear to be partial reads that are filled in by the analysis tool. How can this be valid

data?

Response: We have now included a description of the library preparation, sequencing

and the bioinformatics analysis of the MCF-7 cells in the main text, under the Methods

section (page 12).

4.The validation of the CALU transcript isoforms done by FISH and shown in the

supplement is consistent with mutually exclusive exons. However, the experiment does

not validate use of an alternative promoter. This is an important aspect of the work.

Response: The validation of coupled exons in CALU did not involve the use of

alternative promoter. Moreover, it is extremely challenging to profile TSSs using RNAFISH

due to high GC-content of most first exons. The second challenge is to identify

unique oligos that are suited for such experiment. Therefore, we do not believe the

addition of alternative promoters in RNA-FISH experiment is doable at this point. We

have performed Sanger sequencing to validate additional examples. In the revised

version, we have also performed a comparison between TSSs that were detected in

Encode CAGE data set versus those found by full-length mRNA sequencing (page 4,

paragraph 4; Figure S3 – see response to referee 1, point 2). We find a very strong

concordance between the two experiments and believe that transcript structures

identified in PacBio data are reliable.

5.There is a lot of discussion and motif finding that suggests the activity of an APA

motif, which may be bound by MBNL. Some kind of experimental validation is required

for this, due to the redundancy of motifs bound by the hundreds of RNA binding

proteins expressed by cells.

Response: The most appropriate experiment to assess the role of MBNL in regulating

APA is to perform CLIP-Seq. This would further shed light on the underlying

mechanism for splicing-dependent APA. Our collaborators have made several

attempts to get the CLIP-seq to work in MCF-7 cells but, unfortunately, we have not

been successful in obtaining high-quality data. Thus, we have now removed

speculative discussion points on the role of MBNL from the manuscript.

**Second round of review**

Referee 1 report:

In this revised manuscript, Anvar and colleagues have added methodical detail and clarified general methodological questions. Additional analyses that were requested have been provided, and specific corrections noted have been made. Terminology changes have been made that clear up some confusing statements in the initial version.

The new method detail is sufficient, with one exception - how gene coverage is calculated. For example, in the methods section "Comparison to standard RNA-Seq datasets," the authors state the GENTRAP pipeline was used. The link provided describes a pipeline that can work with multiple aligners and parameters. The authors should state parameters used, so that this is reproducible. These details may reside elsewhere in provided code, but these are salient details that should be included in the methods section. Otherwise, I am satisfied with the new details given.

In general, I feel there are interesting aspects that are not explored or not thoroughly validated, but I do not think this precludes publication of this manuscript. It is an extensive analysis describing an application of a new technology, providing an advance than can be built upon. In sum, analytical issues persist, but the authors describe an incremental step forward for others who are in the process of determining how to best use PacBio long reads.

Some general comments that could improve the manuscript, if so desired:

1. These data were generated in two arms with the second set using distinct chemistry from the first. In addition, there are multiple size selection methods used including some smart cells having no size selection at all. It would be helpful for the authors to speculate how these batch biases might affect their data.

2. Even with the further clarification of the “coupling” method it is still not clear why constitutive exons are excluded from the analysis. I would think some readers would have the same concerns.

3. The majority of the front end of the MCF-7 data processing, QC, and some descriptors about the overall transcriptomic profiles were already done (https://www.pacb.com/blog/data-release-human-mcf-7-transcriptome/). It is unclear at which stage the authors picked up the data. Some clarification is warranted to ensure that data are properly attributed and not duplicated.
